# Organic Fouling Behavior and Mitigation Strategies in Pressure-Driven Distillation

**DOI:** 10.1021/acsestengg.6c00500

**Published:** 2026-09-21

**Authors:** Kian P. Lopez, Hannah T. Cairney, Trisha R. Nickerson, Jacqueline Hall, Lewis C. Salveson, Elizabeth A. Hjelvik, Anthony P. Straub

**Affiliations:** † Department of Chemical and Biological Engineering, 1877 University of Colorado Boulder, Boulder, Colorado 80309, United States; ‡ Department of Civil, Environmental and Architectural Engineering, University of Colorado Boulder, Boulder, Colorado 80309, United States; § Materials Science and Engineering Program, University of Colorado Boulder, Boulder, Colorado 80309, United States; ∥ Department of Mechanical and Process Engineering, ETH Zürich, Zürich 8092, Switzerland

**Keywords:** Pressure-driven distillation, fouling, organic matter, advanced water treatment, water reuse

## Abstract

Pressure-driven distillation (PD) is a membrane-based water treatment process that uses applied pressure to drive vapor transport through a porous hydrophobic membrane. PD offers potential advantages in selectivity and chemical resilience, but its performance with feedwaters containing foulants remains poorly understood. In this study, we examine the organic fouling behavior of PD, isolate the effect of pressure on flux decline, and explore potential fouling mitigation strategies. We find the severity of organic fouling is highly dependent on foulant properties, with more hydrophobic humic acid causing the most severe flux decline and bovine serum albumin proteins causing moderate fouling. Sodium alginate, a hydrophilic gel-forming polysaccharide, showed the least severe fouling despite higher surface coverage. Even after severe flux decline, PD membranes do not show signs of wetting or a loss of rejection. We find that the applied pressure in PD exacerbates fouling by comparing performance in PD to osmotic distillation, an unpressurized analogous process. Finally, we evaluate two fouling mitigation strategies: cleaning with sodium hydroxide, which restores nearly full flux, and continuous direct ozonation, which partially suppresses fouling. Overall, this work provides insights into organic fouling behavior in PD and demonstrates effective fouling mitigation strategies.

## Graphical abstract

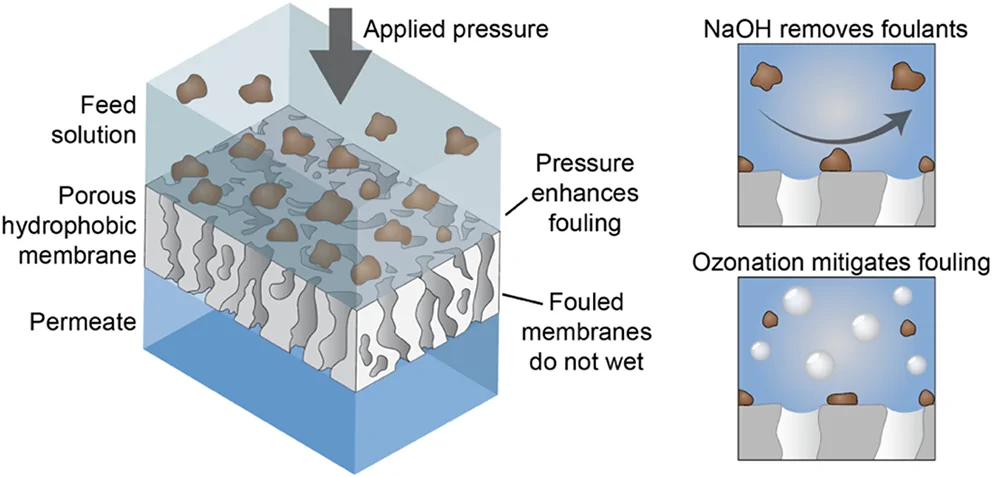

## Introduction

Water treatment processes capable of removing nearly all dissolved constituents are needed to alleviate water scarcity by enabling the production of high-purity water from impaired or nontraditional source waters, while also meeting growing industrial demands for ultrapure water.^1,2^ Pressure-driven distillation (PD) is an emerging membrane-based separation process with the potential to improve advanced water treatment. PD operates by using applied pressure to drive vapor transport through an air-trapping porous hydrophobic membrane.^3,4^ The gas–liquid phase-change facilitates the rejection of nonvolatile solutes. In addition, PD membranes can be fabricated from chemically robust materials that tolerate strong oxidants and extreme pH conditions, enabling cleaning and fouling control strategies that are not feasible in many existing water treatment membrane technologies.^5,6^ Conceptually, PD is similar to thermally driven membrane distillation (MD) processes, but the use of applied pressure, rather than heat, as a driving force makes the process easier to implement for certain applications and reduces energy requirements.^7,8^

PD shows promise for advanced water treatment applications involving feed waters that contain a range of organic foulants, but the impact of these foulants on membrane performance has not been explored. Potential applications of PD include wastewater reuse, ultrapure water production for industrial use, and spaceflight water recovery.^3,9^ Feed waters in these applications contain diverse organic constituents, including natural organic matter, biopolymers, and proteins, at concentrations that induce membrane fouling.^10,11^ Such organic fouling is ubiquitous in membrane-based water treatment and is often a primary cause of performance degradation. In thermally driven MD, for example, organic fouling has been shown to result in flux decline and membrane wetting, with fouling behavior strongly dependent on the chemical nature of the foulant.^12,13^ Unlike MD, PD operates under applied hydraulic pressure and requires membranes with approximately an order-of-magnitude smaller pore sizes than those typically used in thermally driven MD. These conditions are expected to alter foulant deposition, pore blockage, and wetting mechanisms relative to MD, motivating a focused investigation of fouling behavior specific to PD.

In addition to the need to understand organic fouling performance, strategies to mitigate fouling in PD have not been explored. PD membranes can be fabricated from chemically robust hydrophobic materials, such as poly­(tetrafluoroethylene) (PTFE), that have been shown to tolerate exposure to strong oxidants and extreme pH. The use of these conditions for membrane cleaning may expand fouling mitigation options relative to conventional polymeric membranes used in pressure-driven membrane processes. Early work has demonstrated oxidation resistance in PD membrane materials and shown that biofouling can be suppressed using chlorination.^14,15^ However, the use of oxidative chemicals and extreme pH to mitigate fouling in PD has not been comprehensively investigated, and no prior work has evaluated the effect of chemical cleaning on organic fouling in PD. Thus, to develop PD for advanced water treatment there are critical needs to (1) understand organic fouling behavior in conditions relevant to PD and (2) explore fouling mitigation strategies using oxidants and extreme pH.

In this study, we examine organic fouling behavior in PD and demonstrate mitigation using chemical cleaning. Fouling experiments are conducted with three representative foulants: humic acid, sodium alginate, and bovine serum albumin. These model compounds represent classes of organic matter commonly encountered in wastewater reuse, industrial ultrapure water production, and spaceflight water systems, including natural organic matter, polysaccharides associated with extracellular polymeric substances, and proteins. The impact of foulant characteristics on flux decline in PD is examined, and results in PD are compared to unpressurized, osmotically driven experiments to identify the effect of applied pressure on membrane fouling. We then examine the use of sodium hydroxide solutions and continuous ozonation to mitigate fouling, where both cleaning techniques are enabled by the chemical robustness of PD membranes. Overall, this work provides insight into organic fouling behavior and cleaning efficacy in PD, providing foundational knowledge to support the development of PD for advanced water treatment.

## Materials and Methods

### Materials and Chemicals

Commercial poly­(tetrafluoroethylene) (PTFE) membranes (model PTF002LH0P, nominal pore size 20 nm) were obtained from Cytiva and used without further modification. The membranes were hydrophobic and supported on a nonwoven backing layer, as provided by the manufacturer. Humic acid (HA, technical grade), sodium alginate (SA, derived from brown algae), bovine serum albumin (BSA, Fraction V, lyophilized powder), calcium chloride (CaCl_2_), sodium chloride (NaCl), and sodium hydroxide (NaOH) were purchased from Sigma–Aldrich and used as received. Ultrapure water (resistivity ≥18.2 MΩ·cm) produced using a Milli-Q purification system was used for all experimental preparations and cleaning steps.

Feed solutions for fouling experiments were prepared by dissolving each foulant (HA, SA, or BSA) separately at a concentration of 100 mg L^–1^ in ultrapure water containing 3.8 mM CaCl_2_ and 10 mM NaCl to promote foulant aggregation and enable salt rejection measurements. Calcium ions are known to enhance organic fouling through charge screening, cation bridging, and gel strengthening, particularly for humic substances and polysaccharides.^16,17^ NaCl was included as a representative monovalent salt to enable salt rejection measurements during fouling experiments. Flux decline and salt rejection were monitored to assess membrane performance degradation and to identify potential membrane pore wetting.

Total organic carbon (TOC) was measured using a Sievers M5310C TOC analyzer. All fouling solutions were filtered through a 0.45 μm syringe filter prior to use to remove any large particulates. Size exclusion chromatography (SEC) was performed using a Toyopearl HW-50S column with a phosphate buffer containing sodium sulfate as the mobile phase (pH 6.8, 0.1 M ionic strength) at a flow rate of 1 mL min^–1^. Samples were adjusted to match the eluent ionic strength prior to injection. Eluted fractions were monitored for TOC with a Sievers M9 wet-oxidation method TOC analyzer and for UV absorbance (excitation wavelength of 280 nm) with an Agilent 1260 Infinity Series diode array detector.

### Organic Fouling Testing in Pressure-Driven Distillation

Fouling experiments were conducted in a bench-scale dead-end filtration setup at a constant hydraulic pressure of 6.9 bar (100 psi). All experiments were conducted at an applied pressure below the reported liquid entry pressure of 13.8 bar for the 20 nm PTFE membranes used in this study.^14^ All PD experiments utilized a uniform operating temperature of 40 ± 2 °C, which increased the partial vapor pressure difference from the applied pressure across the membrane and facilitated higher, easier to measure fluxes. Flux decline was monitored throughout the experiment, and salt rejection was determined from the feed and accumulated permeate conductivity. Permeate volume was recorded at intervals of 2 to 4 h and converted to volumetric flux. Membranes were run for at least 3 h until flux varied by less than 10% between successive measurements, allowing pressure-induced compaction to stabilize. The stabilized flux was defined as the initial reference flux for subsequent fouling analysis. Circular membrane coupons with a diameter of 2.54 cm, corresponding to an effective filtration area of 5.1 cm^2^, were used for all tests. Feed pressure was supplied using compressed nitrogen gas. The filtration apparatus consisted of a custom-fabricated polycarbonate pressure cell fitted with a membrane support and an O-ring seal to prevent bypass (Figure S1). The fouling solution was then filtered in dead-end mode, and flux was monitored by measuring the change in water volume on the permeate side at intervals of 2 to 4 h. The filtration experiments ran for at least 12 h. The feed side was stirred continuously to reduce concentration polarization. Each fouling test was performed in triplicate with newly prepared feed solutions and separate membrane coupons.

### Osmotic Distillation Experiments

For comparison, osmotic distillation (OD) experiments were performed using the same PTFE membranes and initial solution protocol. A 0.14 M NaCl draw solution was placed on the permeate side of the membrane, while the feed reservoir contained organic fouling solutions. The draw solution concentration was selected to generate a vapor pressure driving force equivalent to that produced by the 6.9 bar applied pressure in PD. The initial average water flux was 95 ± 2 g m^–2^ h^–1^ in PD and 88 ± 6 g m^–2^ h^–1^ in OD, enabling comparison of fouling behavior at matched initial flux. Both compartments were maintained at equal hydrostatic pressures to eliminate any applied pressure differential. Because no pressure is applied in OD, stable flux was reached more quickly; the cell was equilibrated until flux varied by less than 10% between successive measurements, and the stabilized flux was used as the initial reference. The OD system consisted of a glass diffusion cell (PermeGear) with a membrane clamped between the two compartments and an active membrane area of 1.0 cm in diameter (Figure S2). The cell was held at a constant temperature of 40 ± 2 °C using a recirculating water bath. Flux was driven by the vapor pressure gradient across the membrane and monitored by measuring the change in water volume on the draw side of the diffusion cell. Experiments were performed for at least 12 h, with an average duration of 16 h, matching the duration of the PD experiments, and flux decline was normalized to the initial steady-state flux measured in ultrapure water. Differences between conditions were assessed using unpaired two-sample *t*-tests with Welch’s correction for unequal variances. Triplicate tests were conducted for each foulant condition.

### Membrane Cleaning

Following each fouling experiment, membranes were removed and immersed in 0.2 M NaOH solution for 1 h under gentle agitation (150 rpm) to chemically remove deposited organic foulants. Membranes were then rinsed thoroughly with ultrapure water until the rinse pH returned to neutral. Postcleaning water flux was measured under the same conditions used in the initial compaction step. Flux recovery was calculated as the ratio of postcleaning flux to initial pure water flux.

### Static Fouling Experiments

Static fouling experiments were performed using a filtration assembly glass funnel and fritted support base (Figure S3). The membranes were placed between the funnel and support base with the active side of the membrane facing toward the solution. An aluminum clamp was used to seal the assembly system while 200 mL of the fouling solution was in contact with the membrane. The membrane had an effective area of 9.6 cm^2^ in contact with the solution. A shaker table operating at a constant speed of 90 rpm was used to induce hydrodynamic mixing of the fouling solutions in contact with the membrane surface. Under the static fouling conditions, membranes were exposed to the solutions for 96 h. The water permeability and salt rejection of each membrane was then measured in the PD testing apparatus with 3.5 bar applied pressure at 40 ± 2 °C.

### Ozone Experiments

Ozonation experiments utilized a TG-40 ozone generator operating at 15 psi with a flow rate through the generator of 3.0 L min^–1^. Gaseous ozone was diffused directly into the solution for 10 min (Figure S4). Once the ozone generator was turned off, oxygen was continuously diffused into the solution to increase the degradation rate of residual ozone. The residual dissolved ozone concentration was monitored using the Indigo method (described below), and the solution was removed from the system once residual ozone was no longer detectable. For the preozonation scenarios, ozone was diffused in a batch method into 1 L of solution. For direct ozonation scenarios, ozone was diffused into 200 mL of solution in the static fouling assembly immediately following static fouling procedures. The concentration of ozone within the solution was determined using the Indigo Method. In this procedure, 0.15 mL of the ozone exposed solution was mixed with 4 mL of DI water and 0.85 mL of Indigo Reagent II. The absorption of the sample was measured at 600 nm using a UV–vis spectrophotometer (Hach DR6000, CO, USA). Ozone concentration was then calculated using the methods described by Bader and Hoigné.^18^ The ozone concentration was plotted as a function of time to create a concentration curve for each solution (Figure S5). The total ozone exposure, measured as the product of concentration and time (*CT*), was calculated as the area under the concentration curve. For each fouling solution, the target ratio of ozone to TOC was 0.8:1. Given the TOC content of humic acid and sodium alginate, target exposure ranged from 100 to 150 mg·min L^–1^. Static fouling experiments were used in ozonation testing due to the safety and logistical challenges of handling pressurized ozone solutions in our laboratory.

### Membrane Characterization

Membrane morphology and surface coverage were analyzed before and after fouling using scanning electron microscopy (SEM, Hitachi SU8010 microscope) and energy dispersive X-ray spectroscopy (EDX). Samples were sputter-coated with platinum prior to imaging. High-resolution images were taken at multiple locations to assess spatial variability in foulant deposition. Foulant surface coverage was quantified from the SEM images by texture-based segmentation, with the threshold set from pristine membranes.

## Results and Discussion

### More Hydrophobic Foulants Lead to Greater Flux Decline in Pressure-Driven Distillation

Dynamic fouling experiments in PD used three representative organic foulants to capture a range of chemical properties relevant to advanced water treatment applications (Figure 1). Under baseline conditions with an applied pressure of 6.9 bar and a feed solution of 10 mM NaCl and 3.8 mM CaCl_2_, the stable water flux was 95 ± 2 g m^–2^ h^–1^.

**Figure 1:**
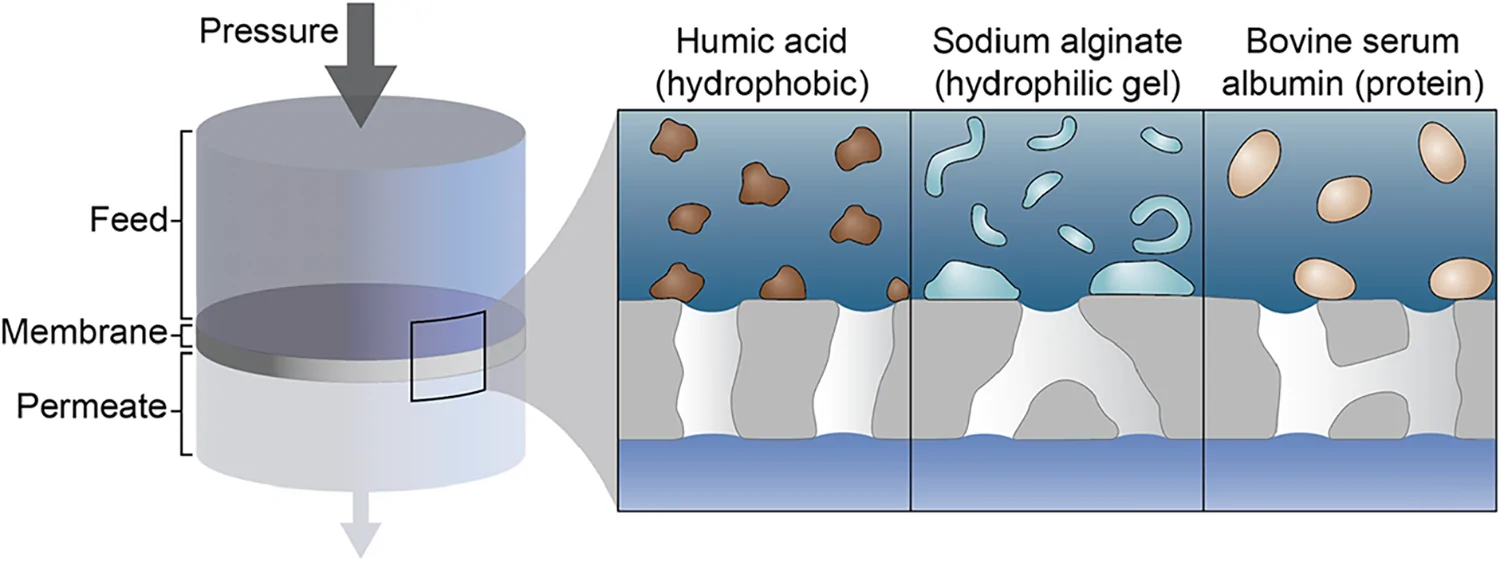
Schematic diagram of pressure-driven distillation (PD) process, where pressure is applied to the feed solution to drive distillation through a porous hydrophobic membrane. Fouling on the membrane from the three organic foulants investigated in this study is illustrated in the inset.

Dynamic fouling experiments showed that the flux decline due to fouling was largely dependent on the nature and hydrophobicity of the foulant. The most rapid and notable flux decline occurred with humic acid (Figure 2A), a hydrophobic foulant representative of natural organic matter commonly present in surface waters and wastewater effluents. Humic acid addition consistently resulted in the most severe flux decline among the foulants tested, causing an 87% decline in water flux (Figure 2B). Fouling with bovine serum albumin, a model protein representative of organic matter present in wastewater, led to a flux reduction of 61%. Sodium alginate, a hydrophilic, gel-forming polysaccharide representative of extracellular polymeric substances produced by microorganisms, resulted in the lowest flux decline of 36% after the same period. Generally, fouling propensity correlated strongly with the foulants hydrophobicity, with the most hydrophobic foulant, humic acid, severely fouling the membrane and the hydrophilic foulant, sodium alginate, causing the least flux decline due to fouling. Similar fouling behavior has been observed in thermally driven MD, where hydrophobic foulants strongly adhere to hydrophobic membrane surfaces.^19,20^ Polysaccharide and protein fouling have also been reported in MD, though none of these behaviors have been measured previously in PD.^21,22^

**Figure 2:**
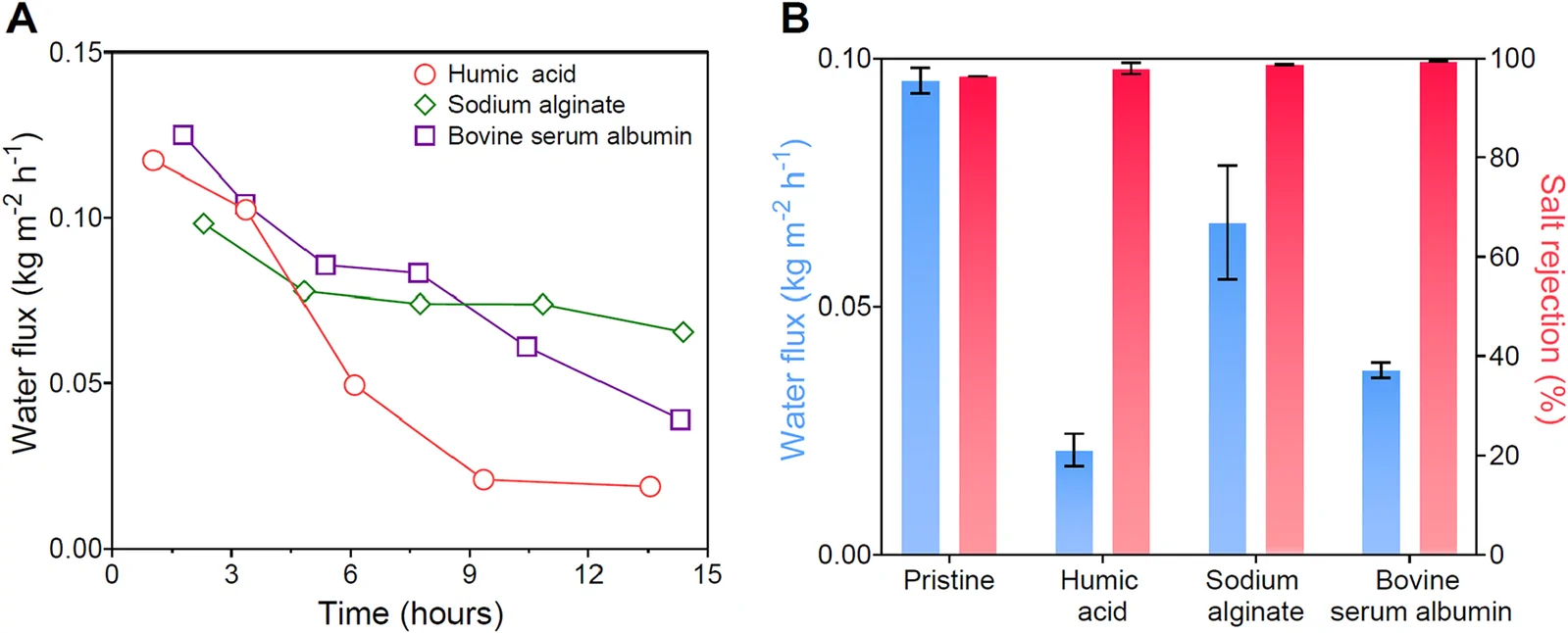
Fouling behavior in pressure-driven distillation for representative organic foulants. (A) Permeation flux as a function of time for humic acid, sodium alginate, and bovine serum albumin fouling solutions. Data points represent time averaged values, and error bars reflect measurement variability within each reporting interval. (B) Water flux and salt rejection for the three representative organic foulants. Error bars denote the mean ± 1 s.d. for measurements from at least three distinct membrane samples. Flux decline is tested at 6.9 bar and a uniform temperature 40 °C using PTFE membranes with a nominal pore diameter of 20 nm. Fouling solutions contained 100 mg L^–1^ organic foulant with 3.8 mM CaCl_2_ and 10 mM NaCl background.

Salt rejection measured on the accumulated permeate after fouling remained between 96–99% across all fouling experiments, indicating that organic fouling primarily increased resistance to water transport rather than inducing membrane wetting or loss of selectivity. Flux also declined steadily throughout, with no increase of the kind that would accompany pore wetting, indicating that the flux decline resulted from increased transport resistance rather than the loss of the vapor gap. Deviations from ideal salt rejection (100%) are attributed to a small, wetted fraction of the membrane area, associated with defects in the commercial PTFE rather than with bulk membrane wetting, and were observed in our prior work with the same membrane in pressure-driven distillation.^14^ The consistently high salt rejection suggests that the air-filled membrane structure remained intact despite substantial flux decline, and that fouling layers formed on the surface did not create continuous liquid channels through the hydrophobic membrane.

SEM imaging confirmed the presence of deposited foulants on the surfaces of all membranes tested (Figure 3). Notably, membranes fouled with sodium alginate exhibited the highest degree of surface coverage (47%, compared with 16% for humic acid and 12% for bovine serum albumin; Figure S6), likely due to the formation of a gel layer resulting from interactions between sodium alginate and calcium ions in the feed solution. This observation aligns with previous findings in the literature, which have demonstrated that calcium ions enhance gelation and deposition of hydrophilic polysaccharides like sodium alginate, contributing to extensive but loosely structured fouling layers.^17,23^ Further SEM imaging of alginate fouling (Figure S7), and EDX analysis (Figure S8) confirmed elevated calcium content within the deposited layer. In contrast, humic acid and bovine serum albumin deposits appeared less uniformly spread across the membrane surface.

**Figure 3:**
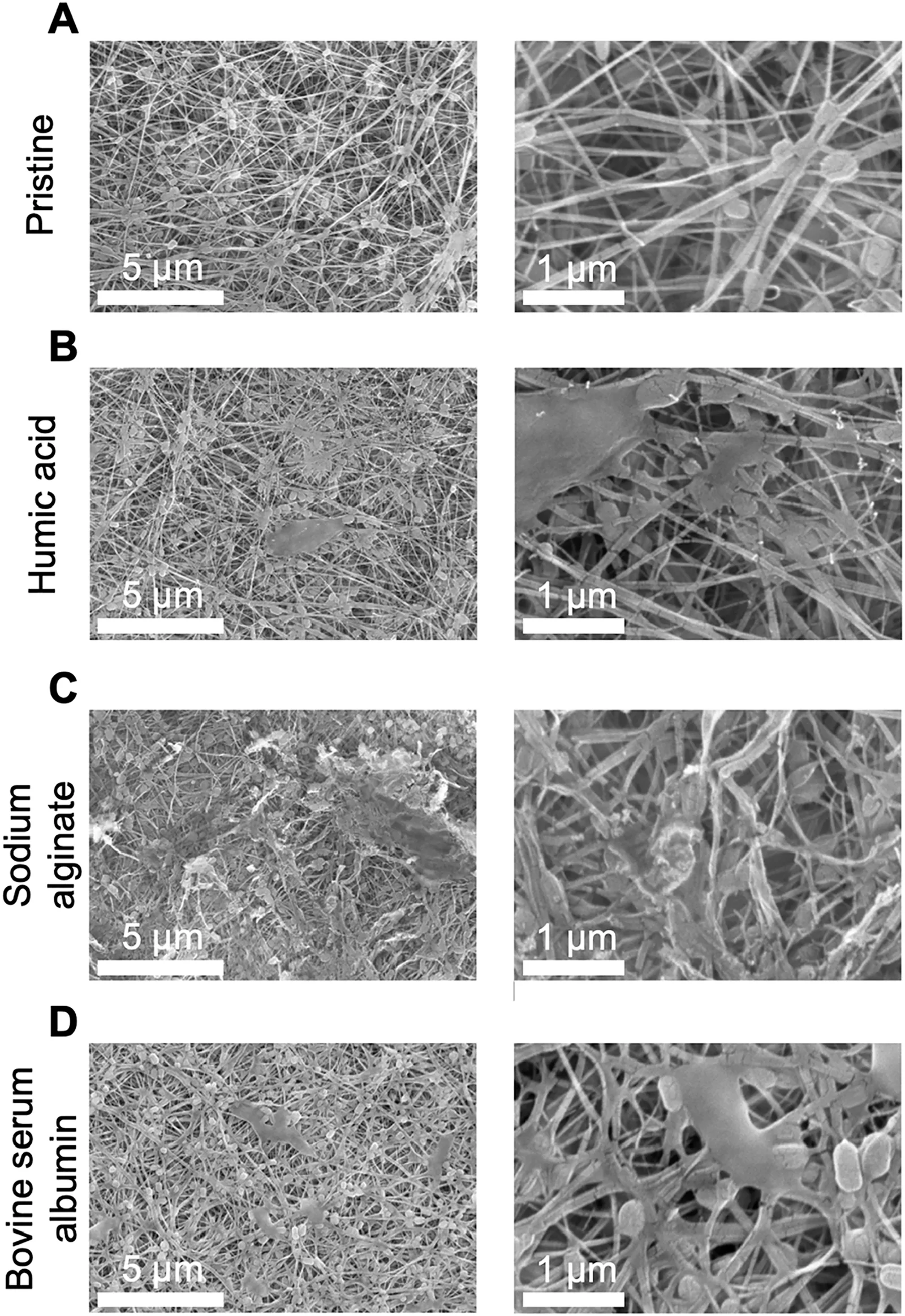
Scanning electron microscopy images of poly­(tetrafluoroethylene) (PTFE) membranes: (A) pristine, (B) fouled with humic acid, (C) fouled with sodium alginate, and (D) fouled with bovine serum albumin. For each condition, low-magnification images are shown on the left and high-magnification images on the right.

Our combined flux decline measurements and membrane characterization reveal distinct differences in fouling behavior that correlate with the physicochemical properties of each organic foulant. Sodium alginate results in the lowest flux decline despite showing the highest surface coverage in SEM imaging. This suggests that flux decline is not governed solely by surface coverage, but by the hydraulic resistance and transport properties of the deposited layer. In the presence of calcium, sodium alginate forms a hydrated gel network that is highly water-rich and likely retains a relatively high water vapor permeability, even when surface coverage is extensive.^24^ In contrast, humic acid, which is more hydrophobic and aromatic, likely forms a denser and less permeable fouling layer that imposes greater resistance to water transport. Bovine serum albumin exhibited intermediate behavior, consistent with protein adsorption and partial compaction at the membrane surface. Despite severe flux decline under exposure to high concentrations of foulants, high salt rejection indicates that fouling primarily increased resistance to water permeability rather than inducing membrane pore wetting. We hypothesize that the small pore size of the PTFE membrane limits foulant penetration into the pore structure, concentrating fouling near the membrane pore entrance and reducing the likelihood of pore intrusion. These observations motivated further experiments to isolate the effect of applied pressure on organic fouling behavior.

### Applied Hydraulic Pressure Exacerbates Fouling in Pressure-Driven Distillation

To isolate the effect of applied hydraulic pressure on membrane fouling, we conducted a parallel set of fouling experiments using osmotic distillation (OD). Unlike PD, which applies pressure to drive vapor transport, OD uses an osmotic pressure gradient generated by a concentrated draw solution (Figure 4A).^25^ Because applied pressure may exacerbate fouling through increased compression and adhesion of foulants, comparing PD and OD under equivalent driving forces provides a controlled basis for assessing pressure-related effects. In these experiments, a 0.14 M NaCl draw solution was used in OD to match the water flux from a 6.9 bar applied pressure in PD.

**Figure 4:**
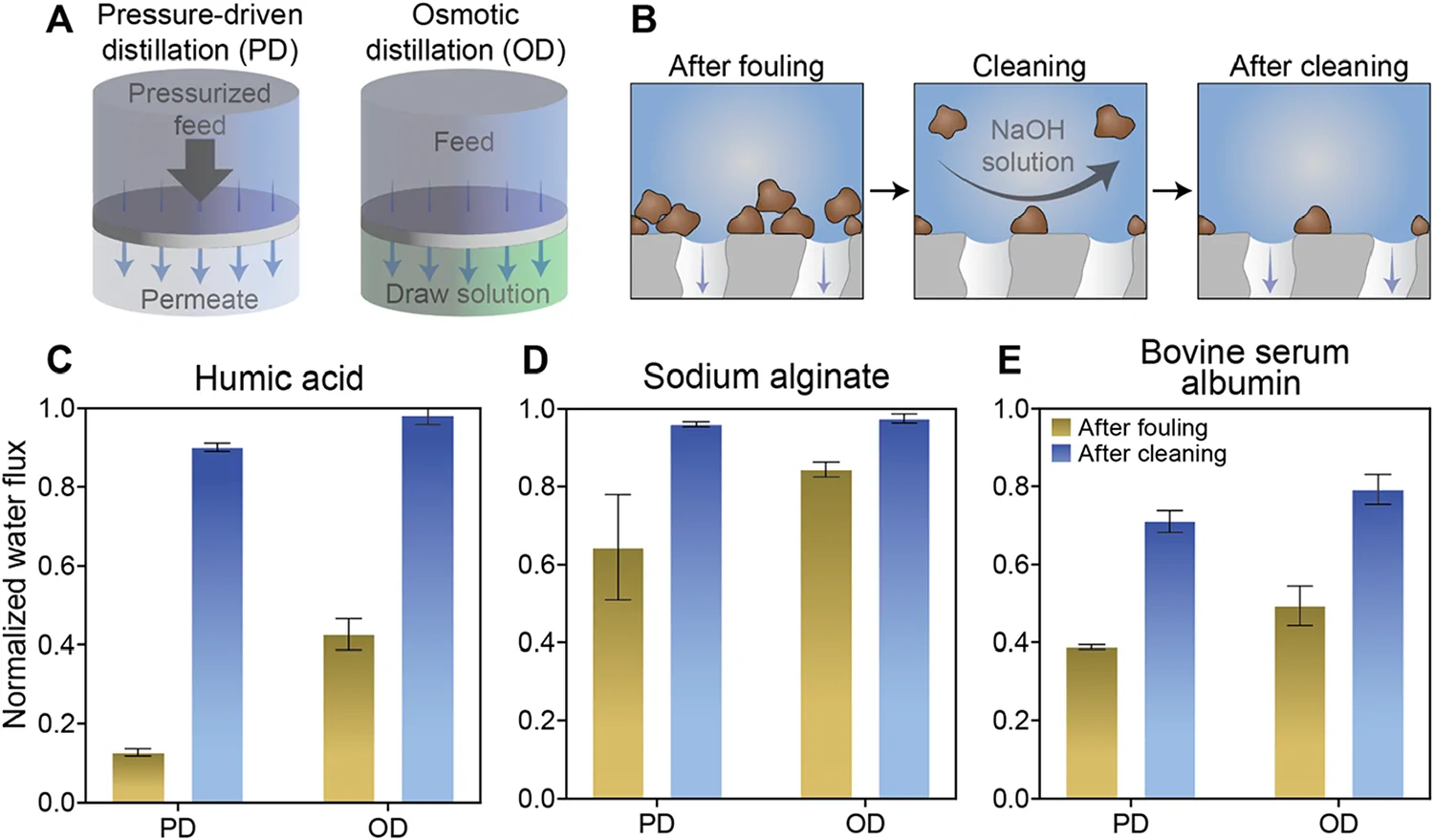
Schematic illustrations of (A) pressure-driven distillation (PD) compared to osmotic distillation (OD) and (B) the cleaning protocol using 0.2 M NaOH to remove organic foulants. Normalized flux in PD and OD after fouling with (C) humic acid, (D) sodium alginate, and (E) bovine serum albumin. Normalized flux after cleaning with 0.2 M sodium hydroxide is also shown. Each foulant was tested at a concentration of 100 mg L^–1^ with 3.8 mM CaCl_2_ and 10 mM NaCl. An applied pressure of 6.9 bar was used for all PD experiments, while a concentration of 0.14 M NaCl was used as the draw solution for all OD experiments. Uniform temperature was maintained at 40 ± 2 °C. Water flux values are normalized relative to the pristine membrane flux. In all plots, error bars denote the mean ± 1 s.d. for measurements from at least three distinct membrane samples.

Flux decline results demonstrate that fouling in OD was consistently less severe than in PD for all tested foulants (Figure 4C–E). For humic acid, flux decline in OD was 57%, compared to 87% in PD, highlighting the exacerbating effect of applied pressure on hydrophobic fouling. Similarly, sodium alginate showed a flux decline of only 16% in OD, whereas PD experienced a 36% reduction. Bovine serum albumin also exhibited reduced fouling under OD conditions, with a 51% decline compared to 61% in PD. These results suggest that the presence of applied hydraulic pressure in PD enhances foulant adhesion or compaction on the membrane surface, leading to more pronounced performance losses. Additionally, salt rejection exceeded 99% in all OD experiments, above the 96 to 99% range observed in PD. Because the compartments were held at equal hydrostatic pressure, there is no driving force for liquid transport through membrane defects.

The comparison between PD and OD reveals that the application of hydraulic pressure increases fouling severity. Across the foulants tested, PD exhibited 20–125% greater flux decline due to fouling than OD under otherwise identical conditions, suggesting that pressure plays a key role in foulant enhanced flux decline. This flux decline was significant for humic acid (*p* = 0.002) and bovine serum albumin (*p* = 0.043), and marginally so for sodium alginate (*p* = 0.063). In PD, hydraulic pressure shifts the liquid–vapor interface deeper into the membrane pores, increasing the extent of liquid water at the pore entrance. As the evaporation interface depresses into the pore, stagnant regions can form near the pore mouth where mixing is limited.^26^ These regions promote local concentration buildup and enhance foulant accumulation at the interface, increasing resistance to water permeability. The applied pressure also compacts the deposited layer itself, reducing its porosity and further increasing its resistance. In contrast, OD operates without an applied hydraulic pressure differential, maintaining a more stable interfacial position and reducing the extent of stagnant water and associated concentration buildup. Both effects apply to any foulant reaching the membrane surface, and the magnitude of pressure-exacerbated fouling depends on the type of foulant. Humic acid exhibited the largest difference between PD and OD, consistent with formation of a denser, higher-resistance deposit under conditions of enhanced interfacial concentration. Bovine serum albumin and sodium alginate showed smaller differences between the two modes. These foulants form more hydrated or less compact layers, and their resistance appears less sensitive to the formation of stagnant regions under the pressures tested.

### Chemical Cleaning is an Effective Fouling Removal Technique for Pressure-Driven Distillation

Membranes used in PD are composed of chemically robust materials, such as PTFE, which can tolerate exposure to strong oxidants and extreme pH.^15^ This chemical resistance enables the use of aggressive cleaning agents to mitigate fouling without compromising membrane integrity. To evaluate the effectiveness of chemical cleaning, fouled PTFE membranes were immersed in a 0.2 M sodium hydroxide (NaOH) solution for 1 h under gentle agitation (Figure 4B). Following the cleaning procedure, membranes were thoroughly rinsed with ultrapure water, and water flux measurements were recorded to assess the extent of performance recovery.

Flux recovery results following chemical cleaning are presented in Figure 4C–E. Flux recovery was evaluated by comparing the postcleaning flux to the initial reference flux for membranes fouled over matched operating times in PD and OD. For membranes fouled with humic acid and sodium alginate, the flux in both PD and OD returned to near-initial values, with greater than 90% flux recovery after cleaning with 0.2 M NaOH, indicating that fouling by these compounds was largely recoverable. Membranes fouled with bovine serum albumin also responded well to chemical cleaning, though flux recovery was slightly lower (approximately 71% for PD and 79% for OD).

Results from the cleaning experiments demonstrate that sodium hydroxide is an effective cleaning agent for membranes fouled by organic compounds, including humic acid, sodium alginate, and bovine serum albumin. Sodium hydroxide facilitates cleaning primarily by hydrolyzing and solubilizing components within the fouling layer, increasing their solubility through enhanced negative charges at elevated pH.^27^ Additionally, sodium hydroxide weakens the fouling layer’s structural integrity by disrupting divalent cation bridges, particularly calcium alginate complexes, through ion exchange, making the fouling layer more porous and easily removed.^16^ Cleaning using sodium hydroxide and other caustic or oxidative solutions offers a promising path for fouling mitigation in pressure-driven separation processes.

### Direct Ozonation Mitigates Flux Decline from Hydrophobic Organic Foulants

Membranes used in PD tolerate exposure to ozone, a strong oxidant frequently used in water treatment.^3,15^ We conducted testing to evaluate the impact of direct ozonation on membrane fouling performance. Such direct ozonation may be possible in wastewater reuse, where ozone is sometimes dosed upstream of membrane treatment.^28,29^ Fouling testing with ozonation was primarily conducted using humic acid since this was the most severe foulant in prior testing. Fouling with direct ozonation was compared to preozonated humic acid and humic acid samples that were not ozonated (Figure 5A). Ozone dosing was calibrated to be equal during preozonation and direct ozonation (Figure S5).

**Figure 5:**
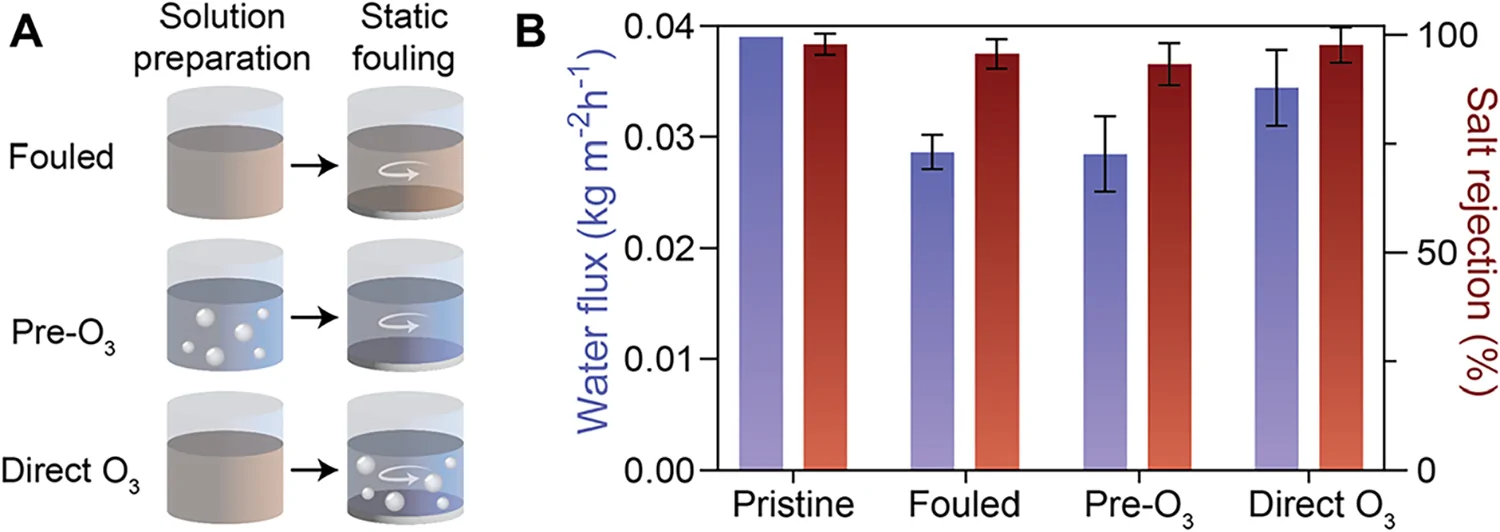
(A) Procedures for static fouling of membranes with ozonation. Membranes were tested after static fouling with untreated foulants, with preozonated foulants, and with direct ozonation during fouling. (B) Water flux (purple) and salt rejection (red) through a poly­(tetrafluoroethylene) (PTFE) membrane in a pressure-driven distillation cell before and after exposure to static fouling, preozonation, and direct ozonation conditions using a 100 mg L^–1^ humic acid solution. The applied pressure was 3.5 bar, and temperature within the system was maintained at 40 °C. Error bars denote the mean ± 1 s.d. for measurements from at least three distinct membrane samples.

Fouling experiments showed that direct ozonation led to a decrease in flux decline due to humic acid fouling (Figure 5B). Prior to fouling, pristine PTFE was found to have consistent flux measurements, approximately 0.039 kg m^–2^ h^–1^ with an applied pressure of 3.5 bar, and high salt rejection, >97.5%, with less than 2.5% variability between flux measurements. Static fouling of the membranes with humic acid resulted in 26% decrease in water flux to 0.029 kg m^–2^ h^–1^. Preozonation of the humic acid solution did not mitigate this decline, with an average flux after preozonation of humic acid of 0.028 kg m^–2^ h^–1^. In contrast, direct ozonation of the membrane during fouling using the same ozone dose helped to mitigate fouling, with an average flux of 0.034 kg m^–2^ h^–1^ and uncompromised salt rejection (>97%). This represents an average recovery of water flux of 50% with some experiments demonstrating up to 80% recovery, although the difference from preozonation did not reach statistical significance (*p* = 0.099). Our results suggest that in situ ozonation is effective at limiting organic fouling and that further improvements may be possible at higher ozone doses, given PTFE’s high oxidative stability.

SEM was used to further understand changes to the membrane surface following the static humic acid fouling and ozonation procedures (Figure S9). Static fouling led to visible accumulation of foulants and pore blockage on the membrane surface. Preozonated membranes also showed extensive fouling. In contrast, directly ozonated membranes displayed reduced surface coverage and greater pore exposure (23% compared with 90% after static fouling and 69% after preozonation, Figure S10), consistent with the observed improvements in water flux.

Contrary to the results with humic acid, ozonation studies using sodium alginate showed that ozonation does not mitigate fouling. Flux decline due to static sodium alginate fouling was approximately 20% whether it was not ozonated, preozonated, or directly ozonated (Figure S11). SEM imaging also did not show mitigation of sodium alginate fouling, with surface coverage of 24% after direct ozonation compared with 7% after static fouling alone, and coverage was highest in the preozonated case at 35% (Figures S12 and S13). This may be because ozonation breaks sodium alginate into lower molecular weight organic species, which can still gel in the presence of calcium and lead to fouling.^30,31^ This hypothesis is supported by total organic carbon (TOC) measurements, which showed 58% reduction in TOC for humic acid after ozonation, but no evident reduction in TOC for sodium alginate (Table S1).

To further evaluate the impact of ozonation on foulant structure and subsequent fouling layer formation, size exclusion chromatography (SEC) was used to characterize humic acid and sodium alginate in feed solutions before and after ozonation (Figure S14). TOC chromatograms derived from SEC revealed that ozonation shifted both humic acid and sodium alginate toward lower molecular weight compounds based on peak shifting toward higher elution volumes (Figure S14A).^32^ This was especially pronounced for alginate which exhibited a pronounced loss of its high-molecular-weight fraction and the emergence of lower-molecular-weight species following ozonation, consistent with depolymerization of the polysaccharide backbone. However, the smaller polysaccharide compounds can still engage in calcium bridging to form gel layers on the membrane, as observed by the lack of fouling mitigation. For humic acid, ozonation also shifted TOC toward lower molecular weights; however, the most significant difference was the reduction of UV absorbance following ozonation (Figure S14B). A reduction in absorbance in the UV-range (250–280 nm) is typically associated with reduced aromaticity.^33–35^ Humic acid is highly aromatic, and hydrophobic interactions cause humic acid molecules to adhere to the hydrophobic membrane and cause fouling.^19,23^ A reduction in aromaticity due to oxidative degradation of humic acid by ozone appears to alleviate membrane fouling by decreasing hydrophobic interactions and increasing hydrophilic oxygen moieties. These results suggest that ozonation partially degrades both foulants but preferentially reduces fouling from hydrophobic organic matter such as humic acid, while lower-molecular-weight alginate fragments remain effective foulants.

Overall, ozonation testing suggests that exposure of PD membranes to ozone reduces fouling from hydrophobic organic matter, such as humic acid, while having limited impact on fouling from hydrophilic organic matter. Prior work has shown that strong oxidation using ozone reduces adhesion of common organic foulants including proteins, polysaccharides, fatty acids, and humic and fulvic acids to hydrophobic surfaces, likely by introducing polar, oxygenated functional groups that render organic matter more hydrophilic.^36,37^ However, fouling from hydrophilic gel-forming sodium alginate may be less affected by ozonation. Notably, sodium alginate caused the least flux decline due to fouling in our testing, suggesting that ozone effectively targets the more problematic foulants for highly hydrophobic PD membranes. Further testing with real feed waters is needed to evaluate whether ozone-based cleaning strategies are feasible for PD membranes under application-relevant conditions.

### Implications

In this study, we investigated the impact of foulant characteristics, applied pressure, and cleaning on organic fouling behavior in PD. We found that fouling behavior was highly dependent on the nature of the foulant where humic acid, a hydrophobic compound, caused the most severe flux decline, while sodium alginate, a hydrophilic gel-forming polysaccharide, resulted in the least flux decline despite showing the highest surface coverage on the membrane. Bovine serum albumin, a model protein foulant, led to moderate flux decline. Across all tests, membranes maintained high salt rejection, even after significant fouling. A direct comparison with osmotic distillation revealed that the application of hydraulic pressure in PD exacerbates fouling, leading to 20–125% higher flux decline under pressure as compared to unpressurized conditions. The magnitude of this effect depended on foulant type, suggesting that applied pressure drives the air–liquid interface deeper into the pore, leading to stagnant regions near the pore entrance that promote local foulant accumulation and increased mass transfer resistance. Pressure also compacts the deposited layer itself, and both effects depend on foulant chemistry and structure. However, chemical cleaning with sodium hydroxide effectively restored flux in membranes, especially those fouled with humic acid or sodium alginate. Continuous direct ozonation of the membrane surface during fouling, which may be possible in advanced water treatment, was also found to reduce flux decline from humic acid fouling.

The observations in this work represent the first systematic evaluation of organic fouling in PD, but further work will be needed to develop a complete understanding of behavior in realistic conditions. A notable limitation of this work was that it required the use of low-flux PTFE membranes with imperfect salt rejection since high performance membranes for PD are not available at a sufficient scale for fouling studies. Future testing should therefore understand how higher flux membrane designs, which have recently been demonstrated, are impacted by membrane fouling.^3^ Furthermore, membranes with omniphobic, Janus, or textured surface properties have shown promising fouling mitigation in thermal MD.^38,39^ Similar designs can also be developed for PD and potentially offer improved fouling performance.^40,41^

This work highlights the potential of chemical cleaning as an effective strategy for mitigating fouling in PD. Future studies should expand the scope of foulants tested and test real feed waters representative of municipal or industrial sources. Such studies will be necessary to assess the broader applicability of the mechanistic insights reported here and to guide the development of fouling mitigation and cleaning strategies under realistic operating conditions. Further optimization of cleaning protocols, including concentration, duration, temperature, and cleaning sequence, will be essential to ensure consistent and efficient membrane recovery. Advancing these research directions will be critical to enabling the practical deployment of PD in large-scale water reuse and industrial water treatment applications and to supporting more sustainable technical solutions to global water scarcity.

## Supplementary Material



## Notes

Experimental apparatus, supplementary membrane characterization, image segmentation analyses, ozonation experiments, SEC and TOC analyses, and additional membrane performance data (PDF)
