# Supplementary material for: Organic Fouling Behavior and Mitigation Strategies in Pressure-Driven Distillation
Source: ACS ES T Eng. 2026 Sep 21;6(10):2961–9. doi: 10.1021/acsestengg.6c00500 (PMC13660393; doi:10.1021/acsestengg.6c00500)
Supplement: Supplementary file 1 [file ee6c00500_si_001.pdf]

Supporting Information for "Organic Fouling Behavior and Mitigation Strategies in Pressure-Driven Distillation"

Kian P. Lopez[a#], Hannah T. Cairney[b#], Trisha R. Nickerson[a], Jacqueline Hall[b], Lewis C. Salveson[b], Elizabeth A. Hjelvik[c], and Anthony P. Straub[b,c,d*]

[a]*Department of Chemical and Biological Engineering, University of Colorado Boulder, Boulder, CO 80309, United States*

[b]*Department of Civil, Environmental and Architectural Engineering, University of Colorado Boulder, Boulder, CO 80309, United States*

[c]*Materials Science and Engineering Program, University of Colorado Boulder, Boulder, CO 80309, United States*

[d]*Department of Mechanical and Process Engineering, ETH Zürich, Zürich 8092, Switzerland*

# These authors contributed equally to this work
* Corresponding author, E-mail: astraub@ethz.ch

Table of Contents

Figure S1. Pressure-driven distillation module 3

Figure S2. Diffusion cell for osmotic distillation experiments....................3

Figure S3. Static fouling system 4

Figure S4. System setup for pre-ozonation of a stock fouling solution and direct ozonation 5

Figure S5. Aqueous ozone concentration for pre- and direct ozonation experiments 6

Figure S6. Foulant surface coverage on PTFE membranes quantified by texture-based image segmentation. 7

Figure S7. Scanning electron micrographs of PTFE membranes fouled by sodium alginate 8

Figure S8. Energy dispersive X-ray spectroscopy (EDX) of sodium alginate fouling layer...........9

Figure S9. Scanning electron micrographs of PTFE membranes following exposure to humic acid static fouling, pre-ozonation with static fouling, and static fouling with direct ozonation....10

Figure S10**.** Humic acid surface coverage for pristine, statically fouled, pre-ozonated, and direct ozonated PTFE membranes quantified by texture-based image segmentation. 11

Figure S11. Water flux and salt rejection of membranes after sodium alginate fouling and ozone treatment. 12

Figure S12. Scanning electron micrographs of PTFE membranes following exposure to sodium alginate fouling, pre-ozonation with static fouling, and static fouling with direct ozonation 13

Figure S13. Sodium alginate surface coverage for pristine, statically fouled, pre-ozonated, and direct ozonated PTFE membranes quantified by texture-based image segmentation 14

Figure S14. Size exclusion chromatography chromatograms of humic acid and sodium alginate before and after ozonation. 15

Table S1. Total organic carbon, inorganic carbon, and total carbon in the humic acid and sodium alginate solutions before and after exposure to ozone 16

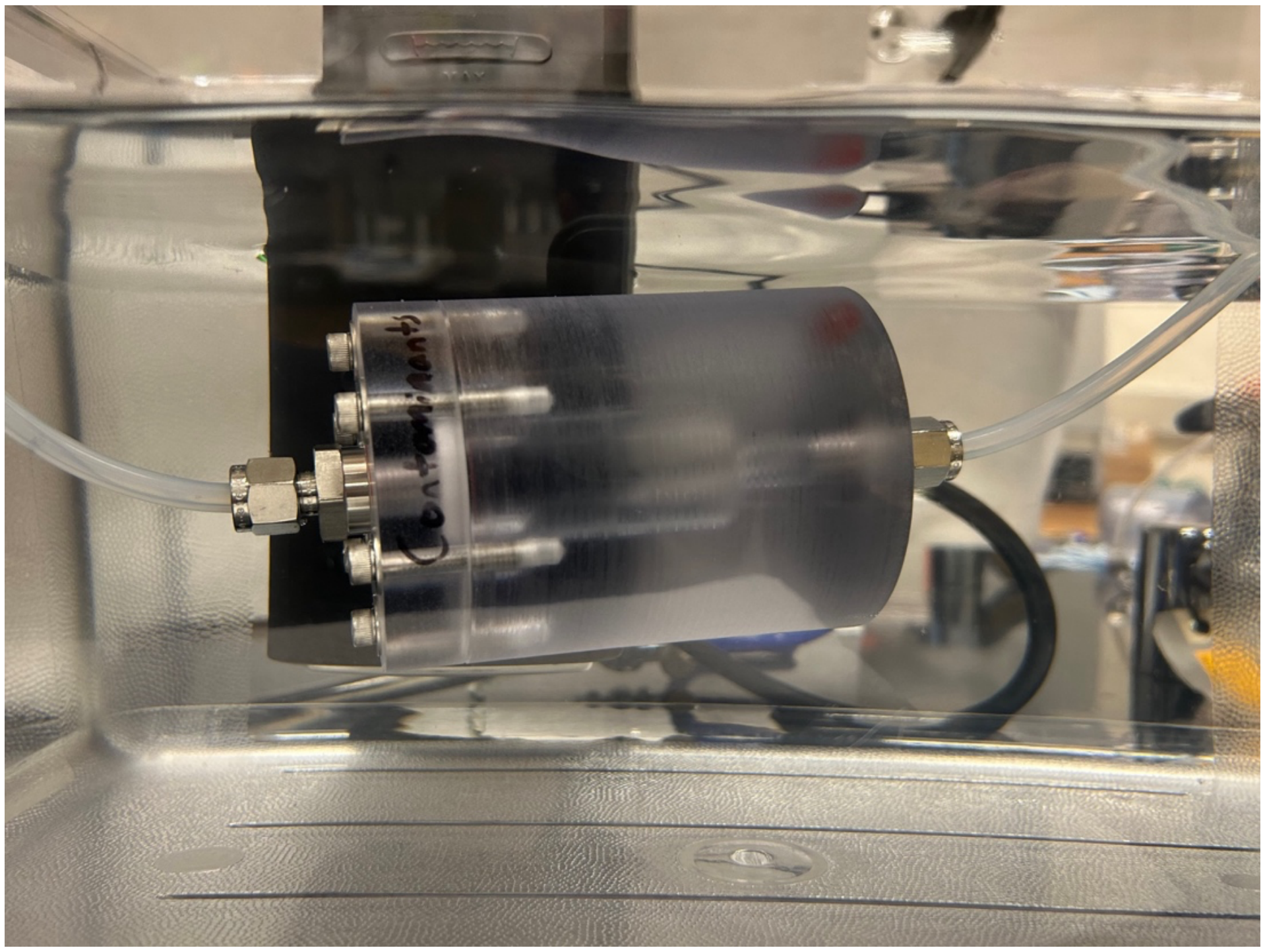

Figure S1. Pressure-driven distillation module used for performance testing. The module was equipped with a magnetic stir bar for mixing and submerged into a water bath to regulate temperature to 40 ± 2 °C.

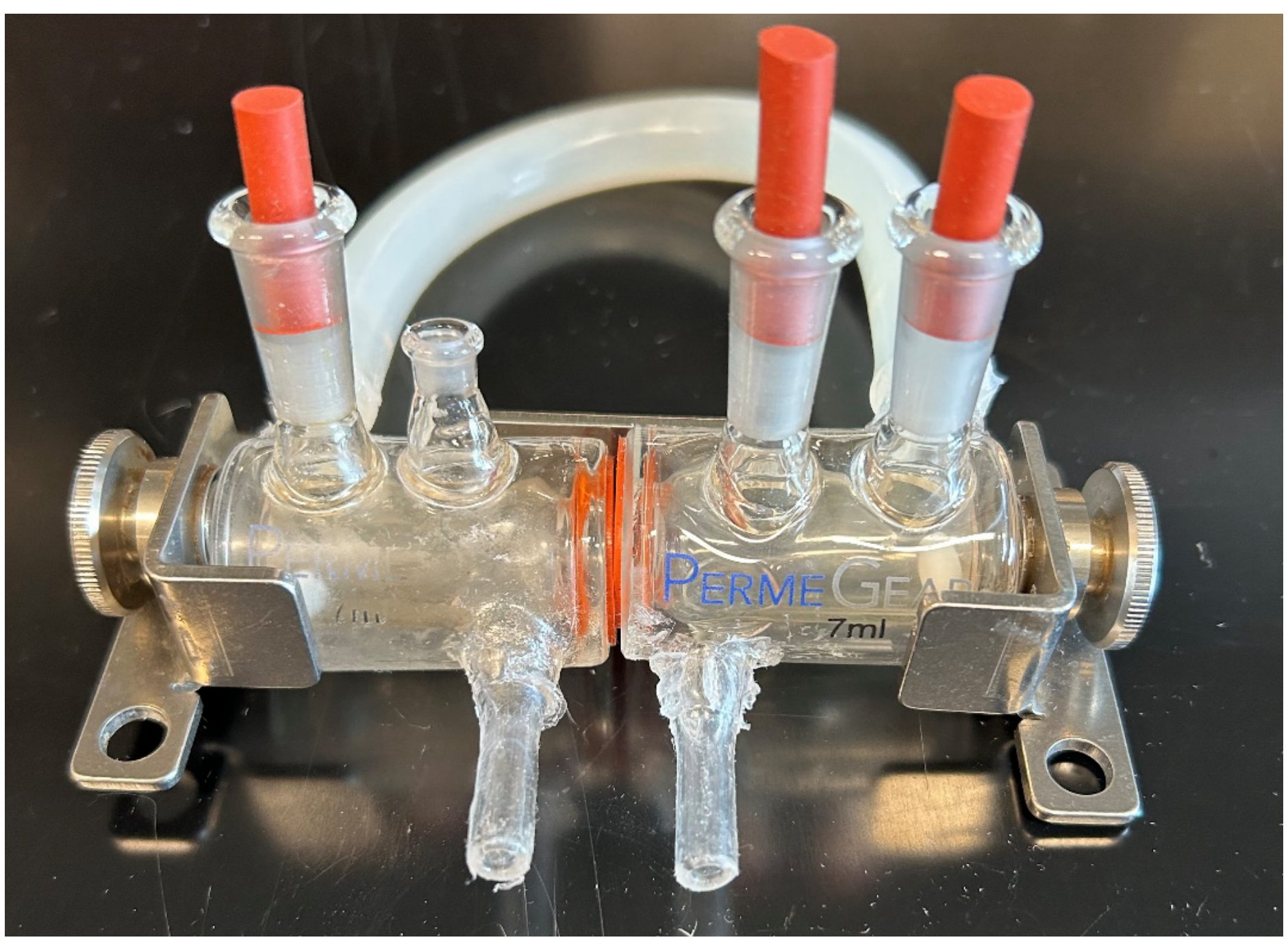

Figure S2. Glass diffusion cell used for osmotic distillation experiments. The shell side of the diffusion cell was used to regulate temperature to 40 ± 2 °C.

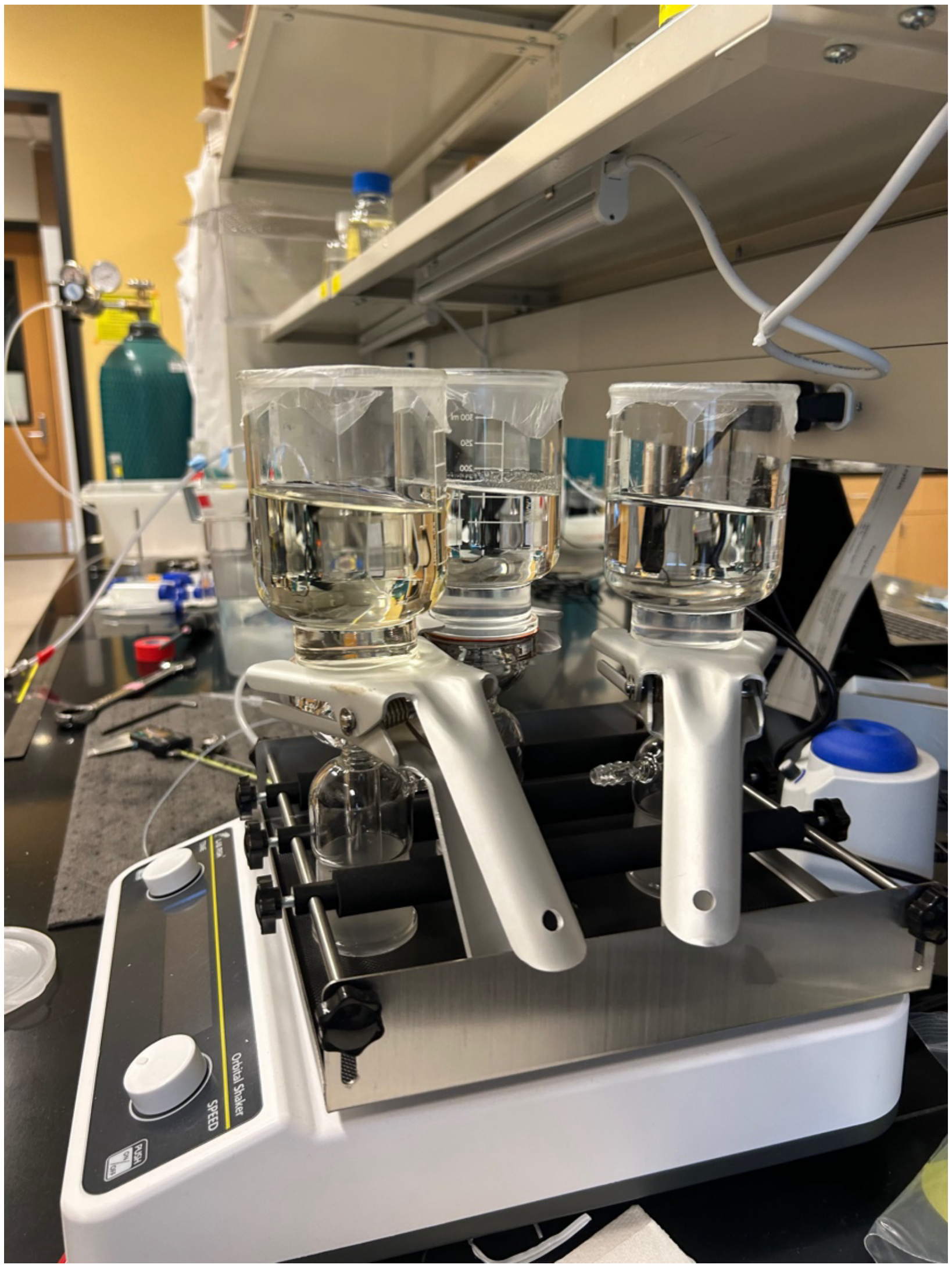

Figure S3. Static fouling setup used in ozonation experiments. Above setup features three simultaneous static fouling experiments with 200 mL of each fouling solution.

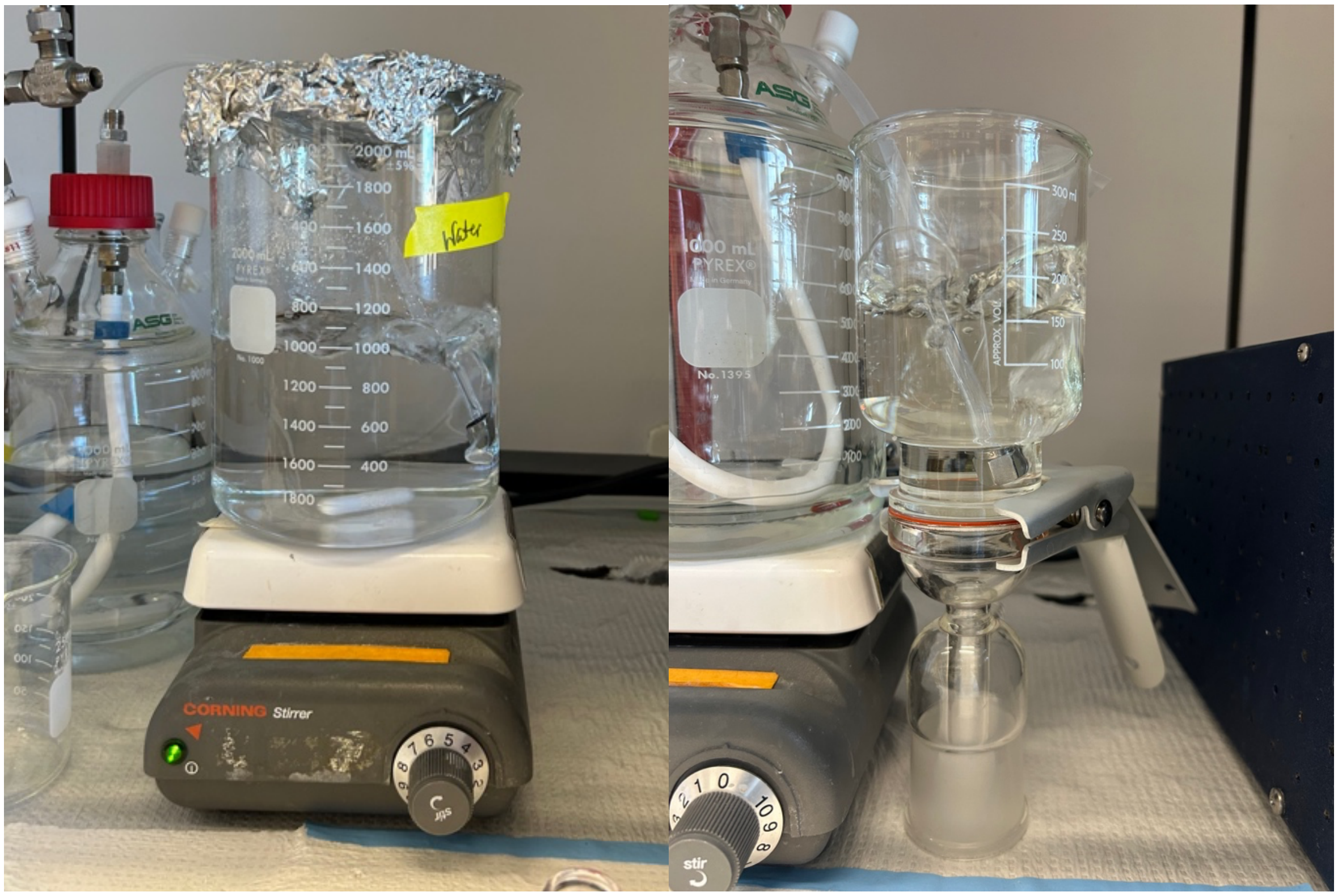

Figure S4. System setup for pre-ozonation of a stock fouling solution (left) and direct ozonation in contact with the fouling solution and membrane surface (right).

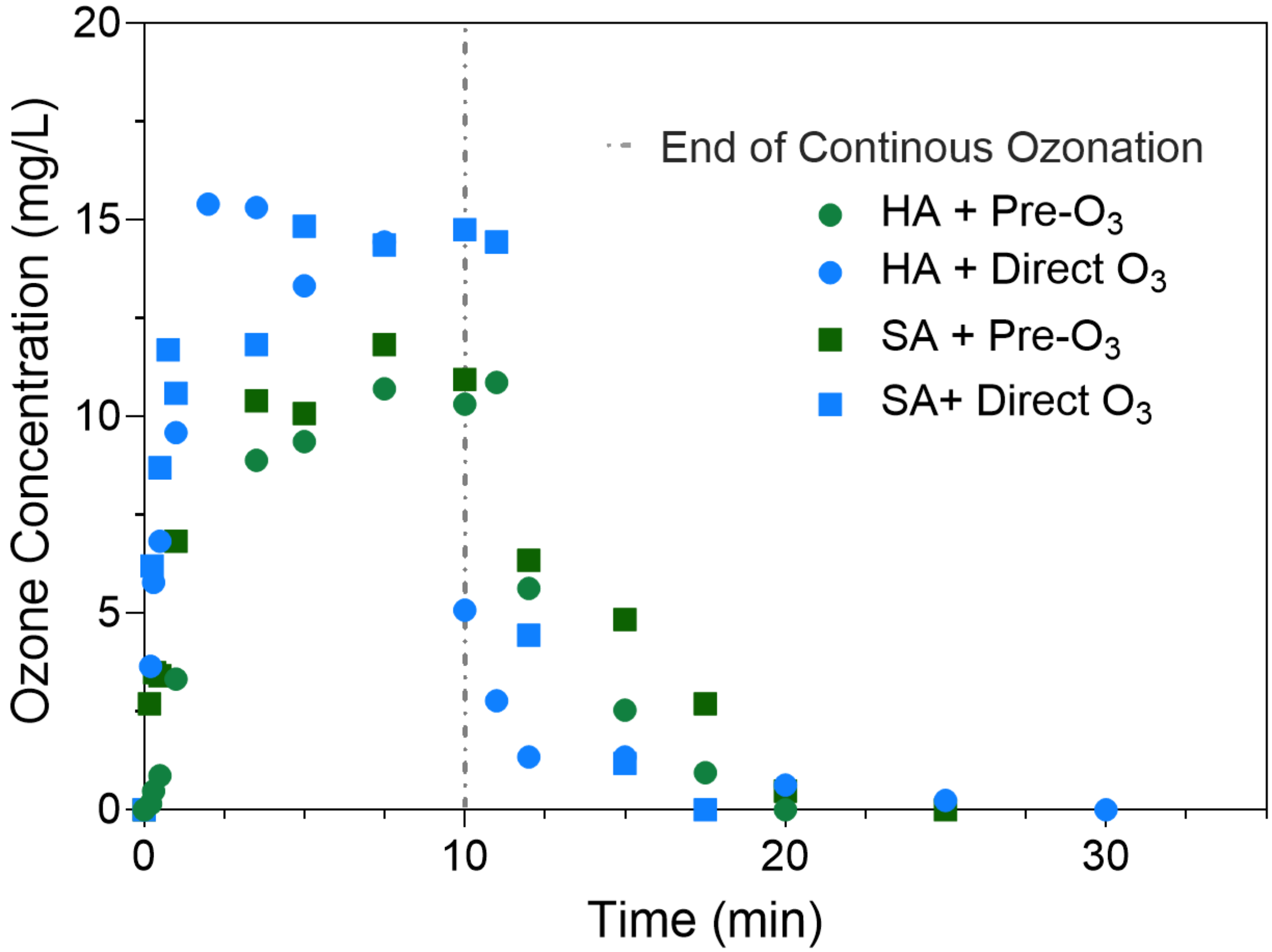

Figure S5. Aqueous ozone concentration versus time during pre-ozonation with humic acid (green circle), direct ozonation with humic acid (blue circle), pre-ozonation with sodium alginate (green square), and direct ozonation with sodium alginate (blue square). Concentration was measured using the Indigo Method. Ozone generation was ended after a continuous flow for 10 minutes. Concentration was measured until the ozone residual was no longer detected in solution.

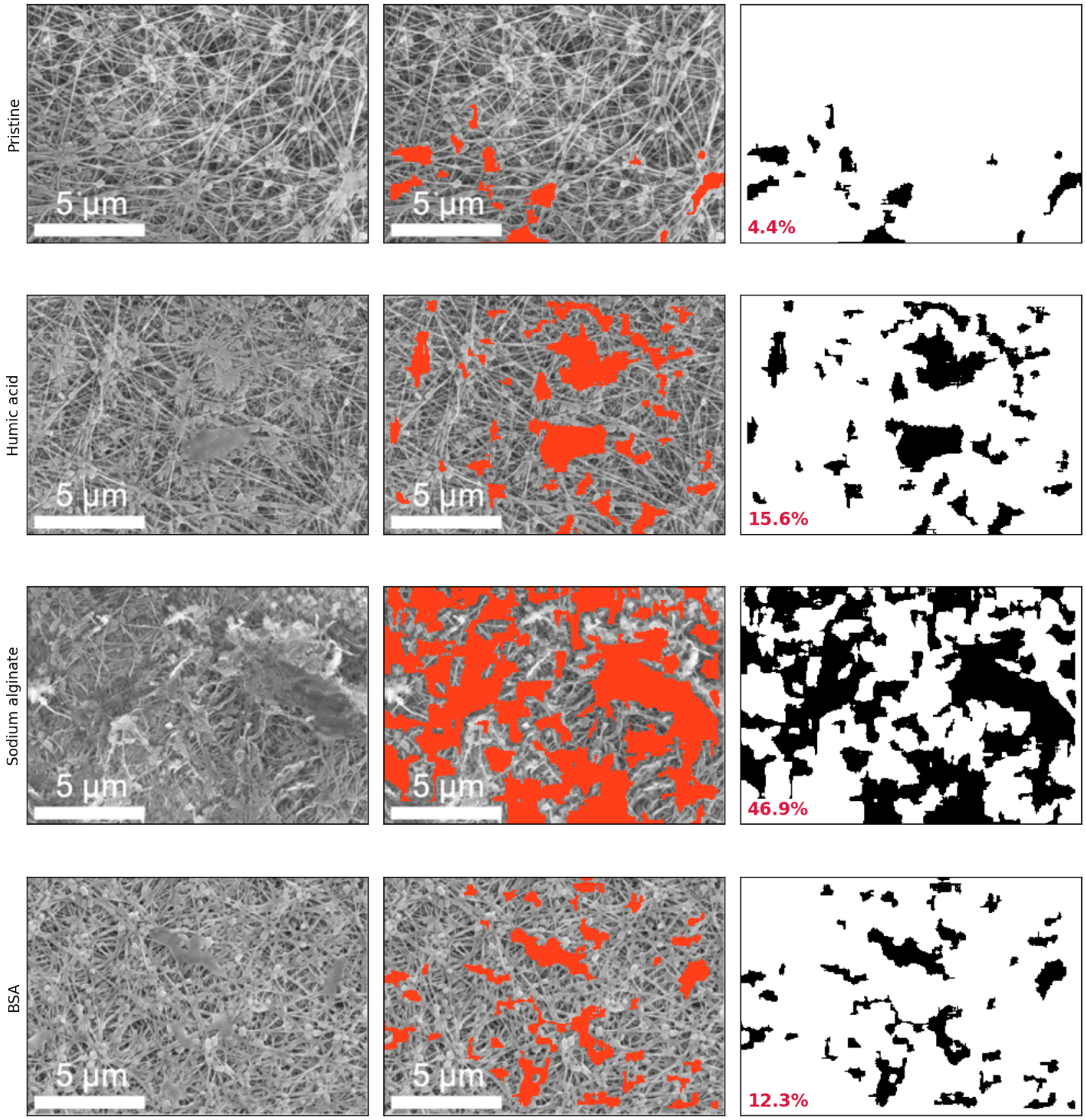

Figure S6. Foulant surface coverage on PTFE membranes quantified by texture-based image segmentation. Left: scanning electron micrographs. Center: segmented deposits overlaid in orange. Right: binary masks with the corresponding fouled area fraction.

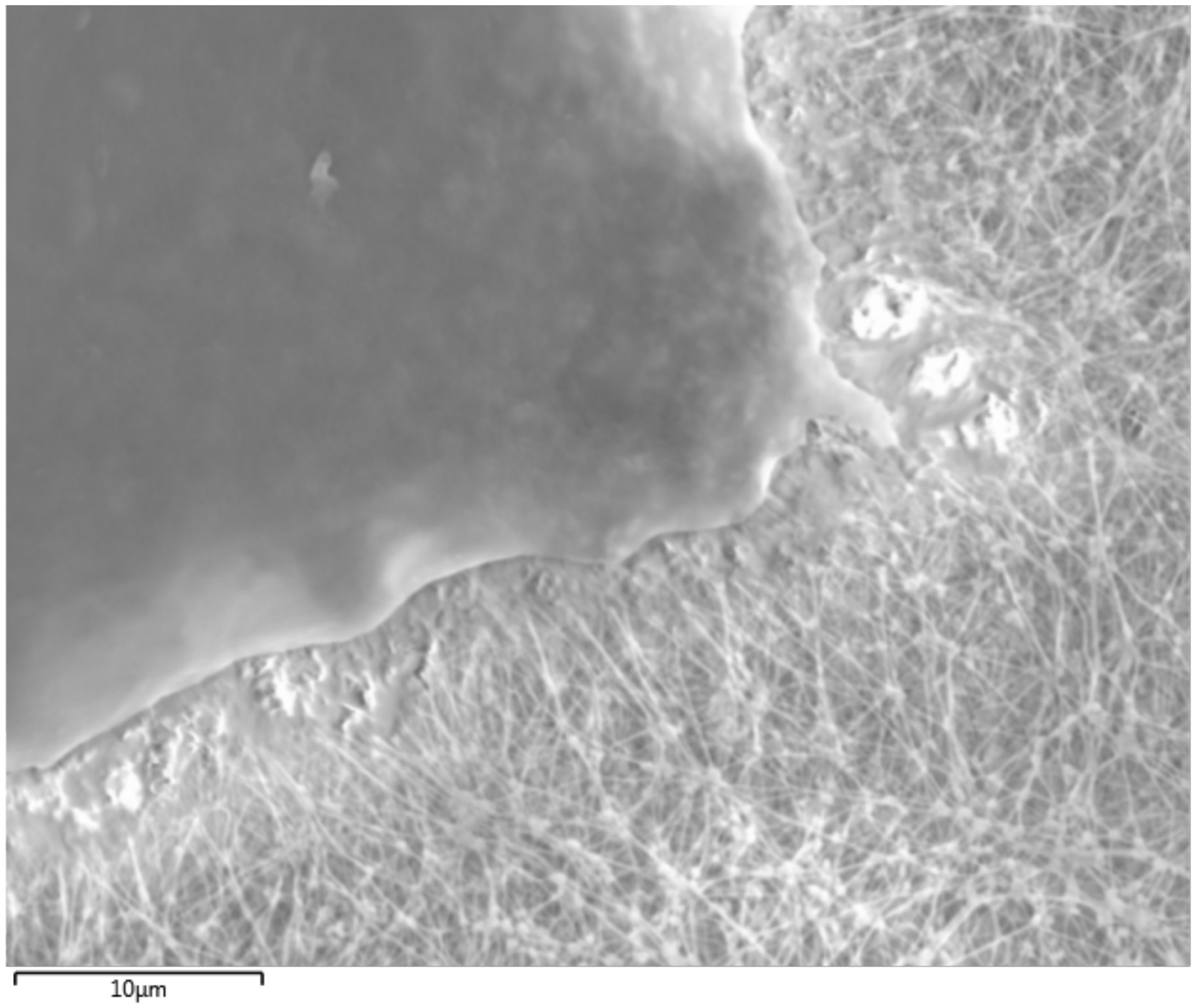

Figure S7. Scanning electron micrograph of a PTFE membrane following exposure to sodium alginate static fouling.

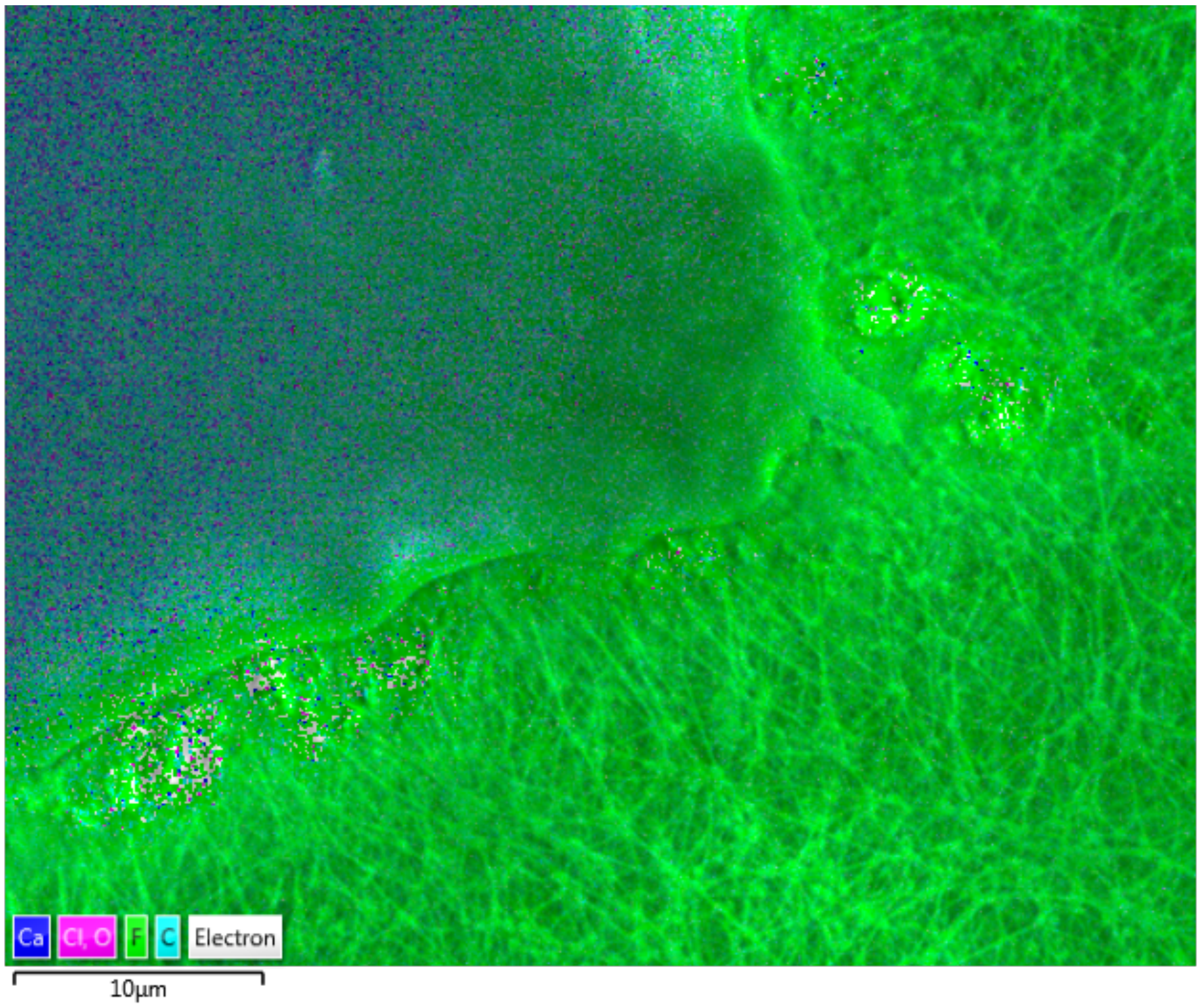

Figure S8. Energy dispersive X-ray spectroscopy (EDX) of a PTFE membrane following exposure to sodium alginate fouling.

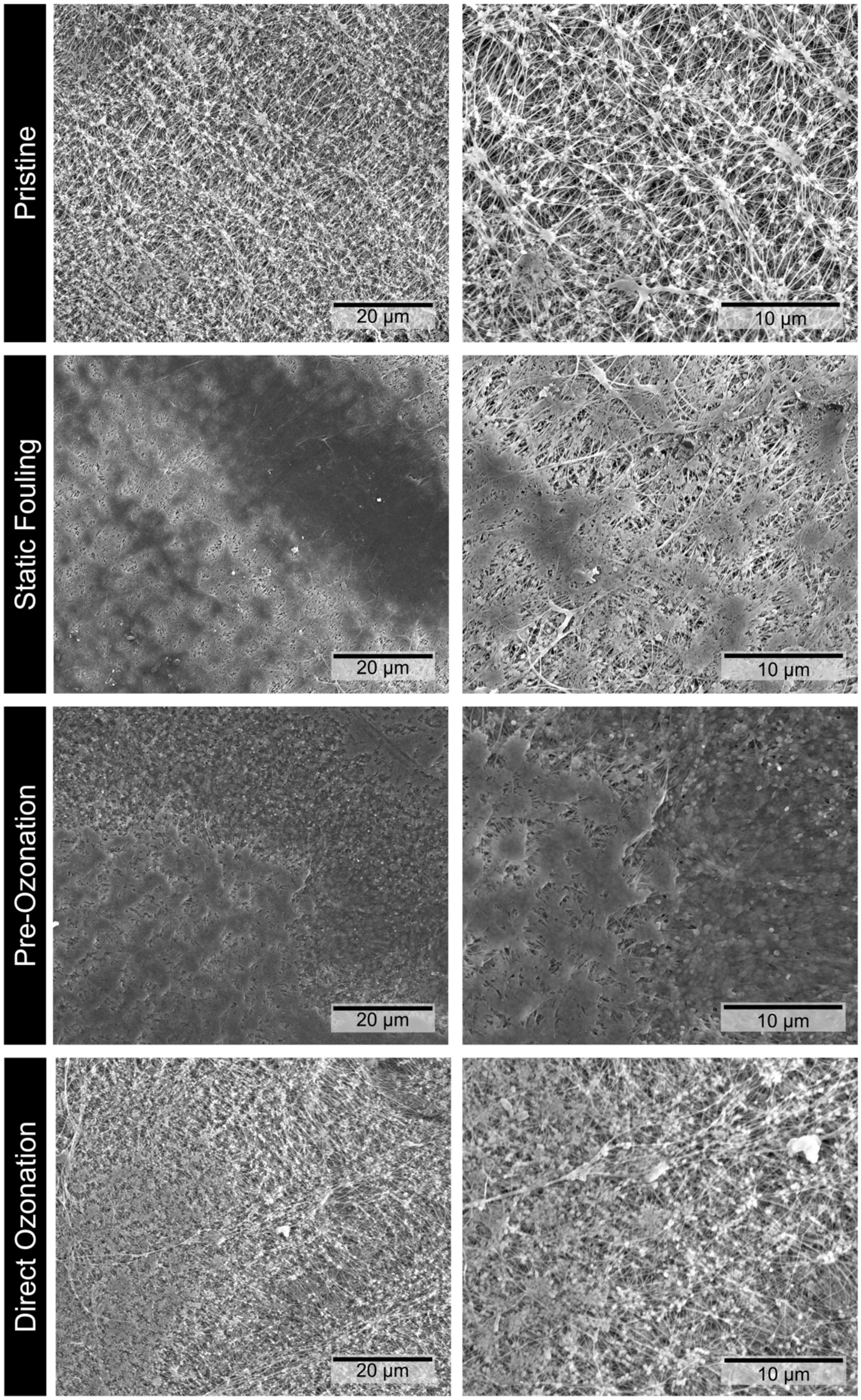

Figure S9. Scanning electron micrographs of PTFE membranes following exposure to humic acid static fouling, pre-ozonation with static fouling, and static fouling with direct ozonation.

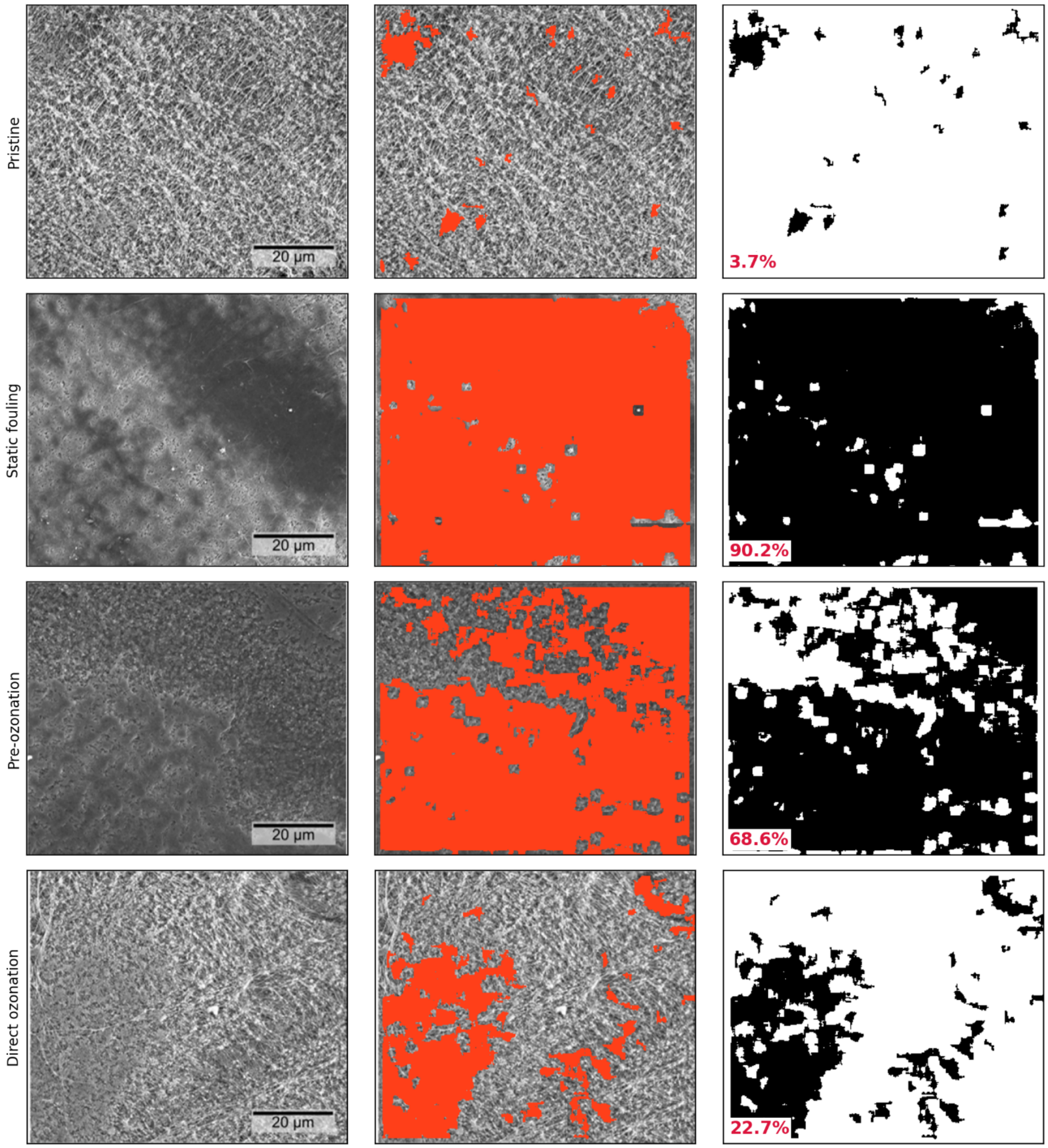

Figure S10. Humic acid surface coverage for pristine, statically fouled, pre-ozonated, and direct ozonated PTFE membranes quantified by texture-based image segmentation. Left: scanning electron micrographs. Center: segmented deposits overlaid in orange. Right: binary masks with the corresponding fouled area fraction.

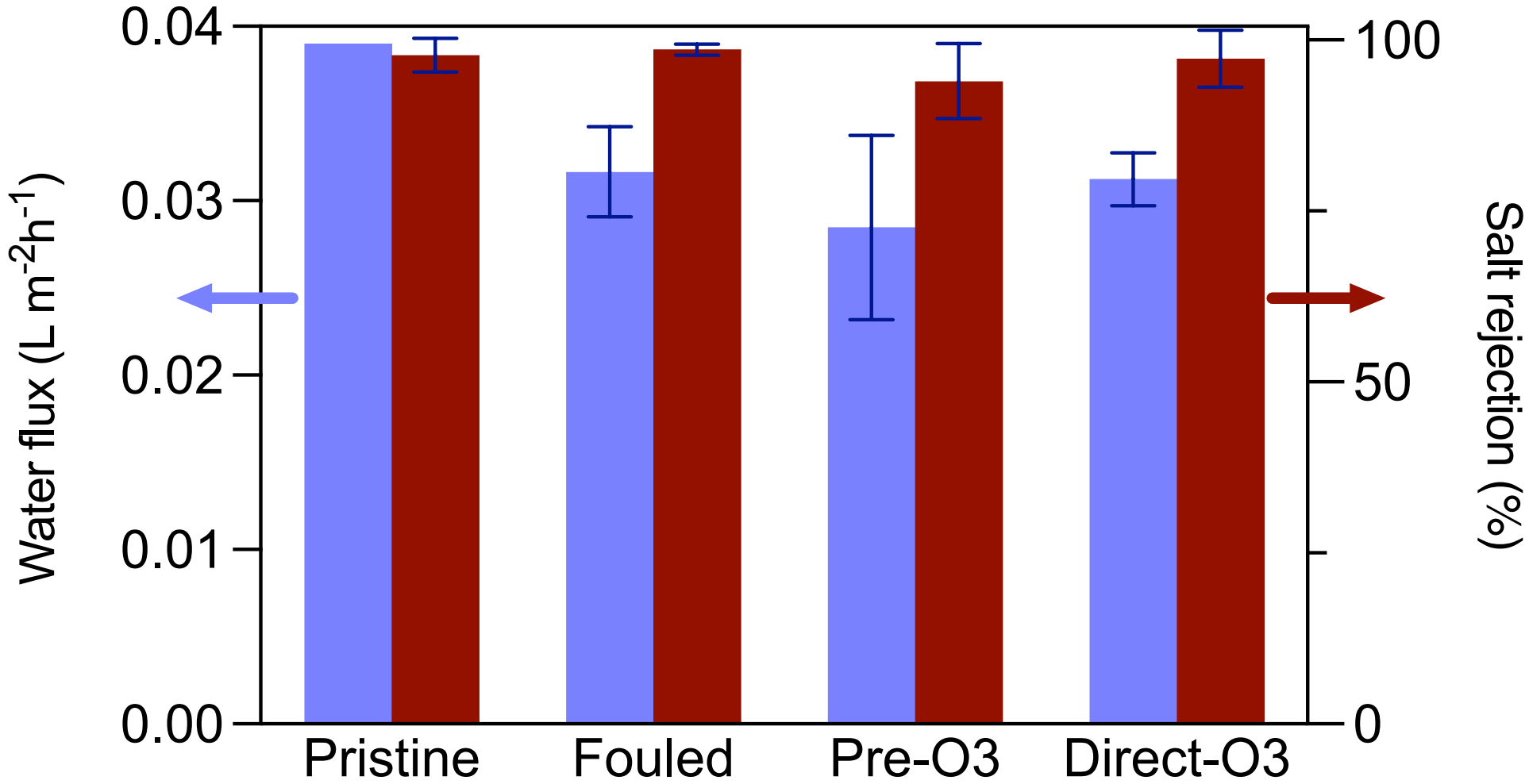

Figure S11. Water flux (purple) and salt rejection (red) through a PTFE membrane in pressure-driven distillation before and after exposure to static fouling, pre-ozonation, and direct ozonation conditions using a 100 mg L^{-1} sodium alginate solution. The applied pressure was 3.5 bar, and temperature within the system was maintained at 40 °C. Error bars denote the mean ±1 s.d. for measurements from at least three distinct membrane samples.

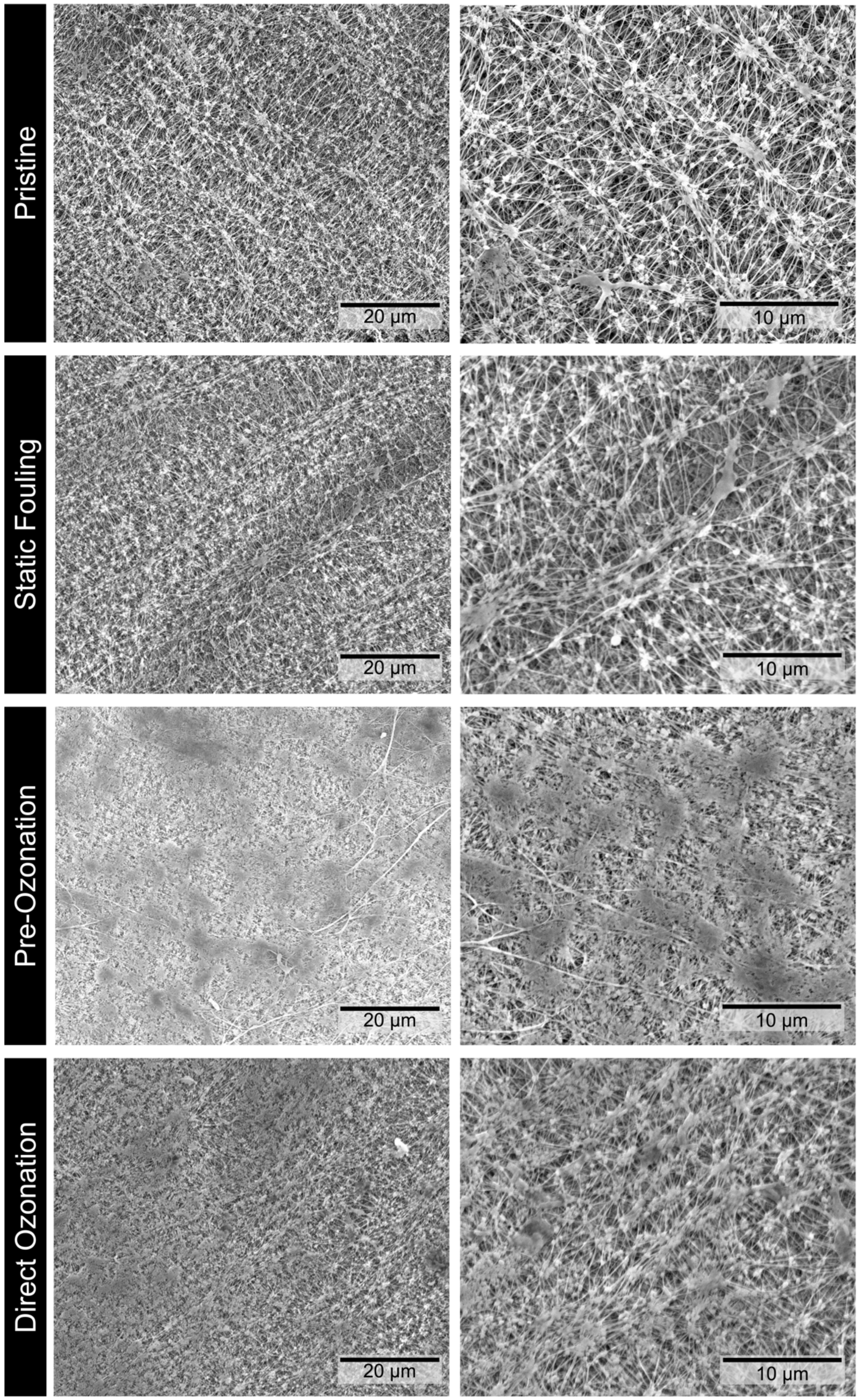

Figure S12. Scanning electron micrographs of PTFE membranes following exposure to sodium alginate static fouling, pre-ozonation with static fouling, and static fouling with direct ozonation.

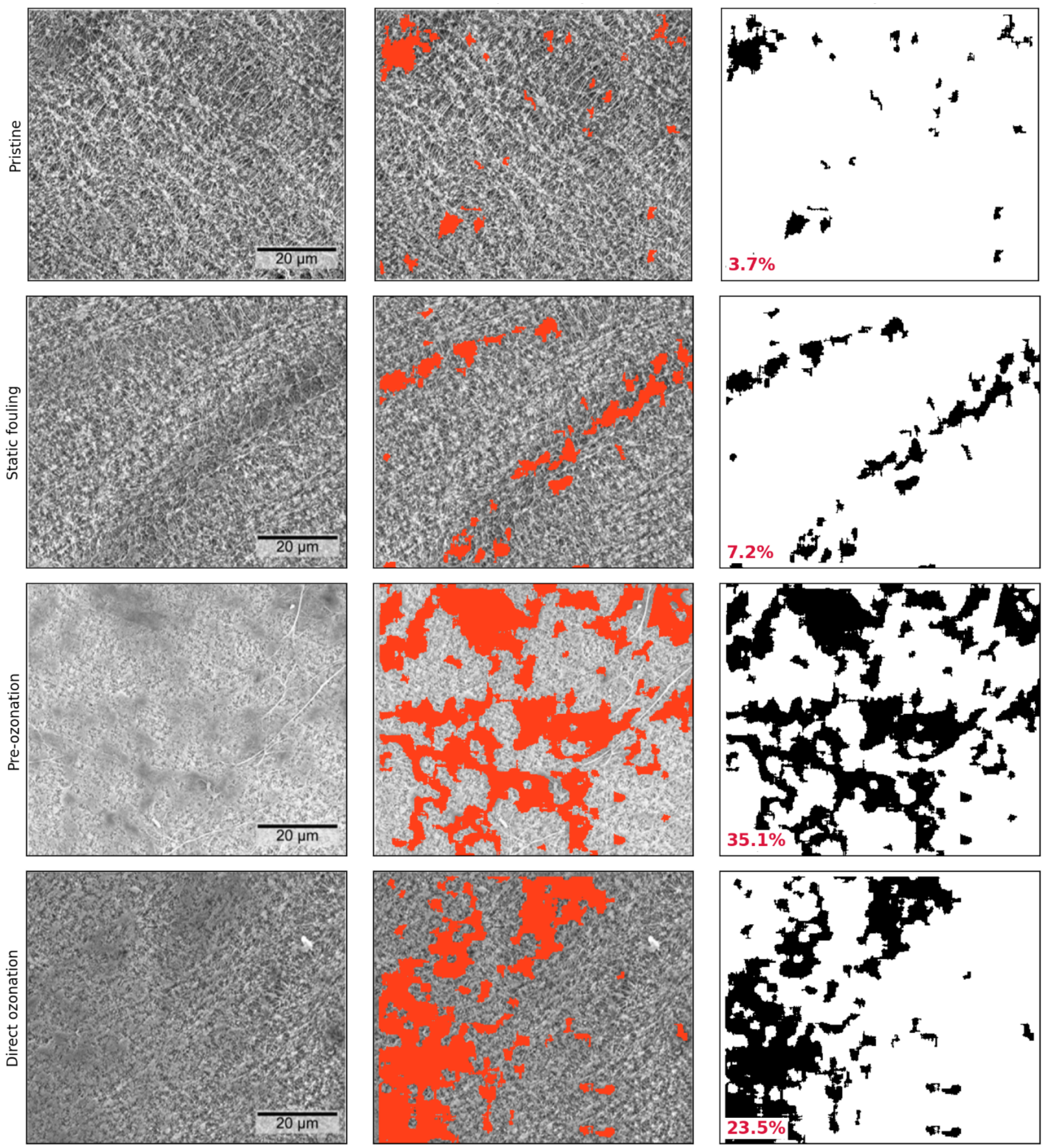

Figure S13. Sodium alginate surface coverage for pristine, statically fouled, pre-ozonated, and direct ozonated PTFE membranes quantified by texture-based image segmentation. Left: scanning electron micrographs. Center: segmented deposits overlaid in orange. Right: binary masks with the corresponding fouled area fraction.

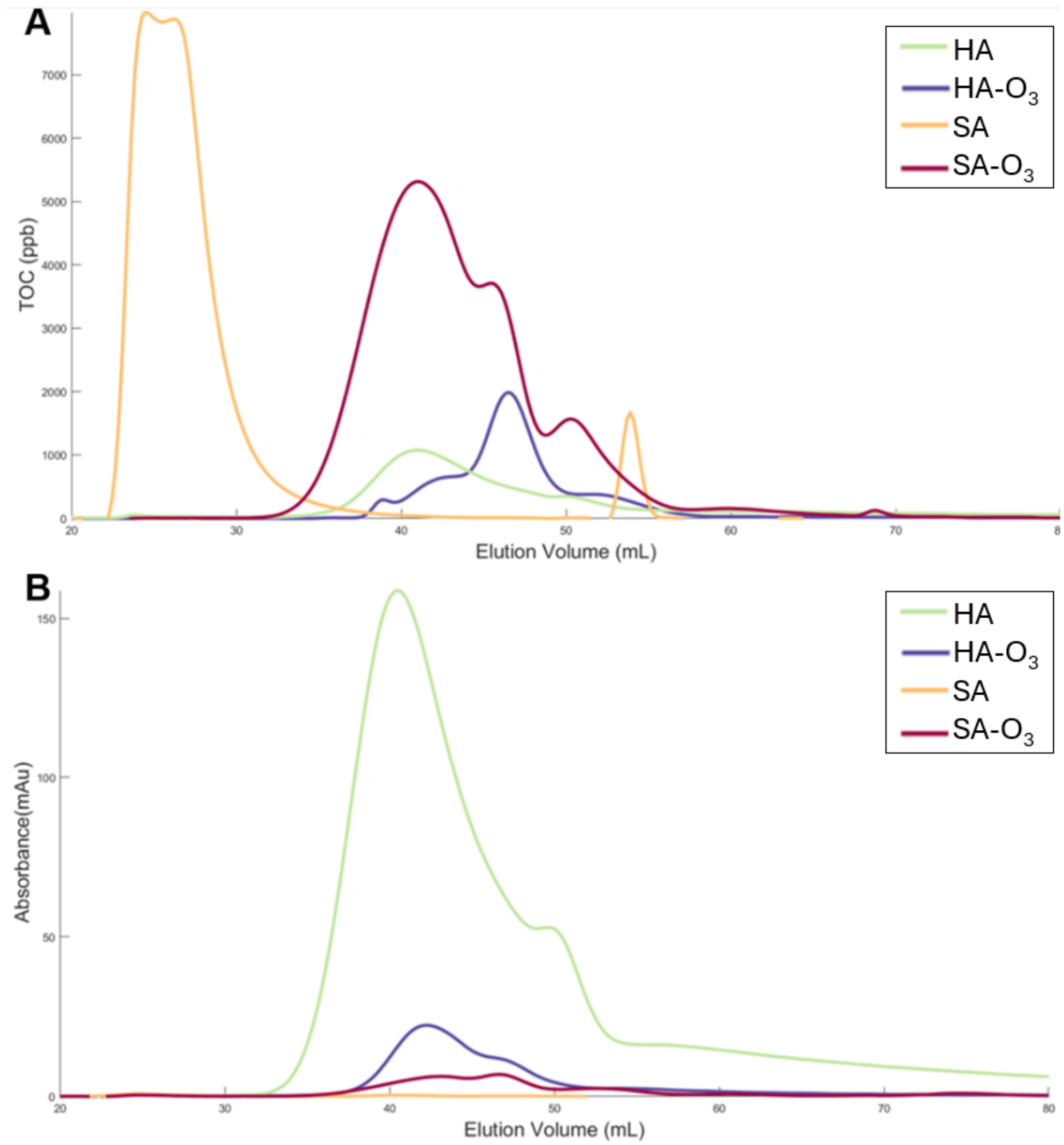

Figure S14. Size exclusion chromatography (SEC) chromatograms of humic acid (HA) and sodium alginate (SA) before and after ozonation (O_3). (A) Total organic carbon (TOC) and (B) UV absorbance at an excitation wavelength of 280 nm.

Table S1. Total organic carbon in the humic acid and sodium alginate solutions before and after exposure to ozone for 10 min and the percentage reduction of each carbon type.

Foulant	Condition	Total Organic Carbon (mg/L)
Humic Acid	Stock	3.41±0.27
	Ozonated	1.42±0.01
	% Reduction	58.36%
Sodium Alginate	Stock	8.78±1.08
	Ozonated	8.67±0.61
	% Reduction	1.25%
